# 2,3,7,8-Tetrachlorodibenzo-*p*-dioxin abolishes circadian regulation of hepatic metabolic activity in mice

**DOI:** 10.1038/s41598-019-42760-3

**Published:** 2019-04-24

**Authors:** Kelly A. Fader, Rance Nault, Claire M. Doskey, Russell R. Fling, Timothy R. Zacharewski

**Affiliations:** 1 Department of Biochemistry & Molecular Biology, Michigan State University, East Lansing, MI 48824 USA; 2 Institute for Integrative Toxicology, Michigan State University, East Lansing, MI 48824 USA; 3 Department of Microbiology and Molecular Genetics, Michigan State University, East Lansing, MI 48824 USA

**Keywords:** Metabolomics, Transcriptomics, Metabolic disorders

## Abstract

Aryl hydrocarbon receptor (AhR) activation is reported to alter the hepatic expression of circadian clock regulators, however the impact on clock-controlled metabolism has not been thoroughly investigated. This study examines the effects of AhR activation on hepatic transcriptome and metabolome rhythmicity in male C57BL/6 mice orally gavaged with 2,3,7,8-tetrachlorodibenzo-*p*-dioxin (TCDD) every 4 days for 28 days. TCDD diminished the rhythmicity of several core clock regulators (e.g. *Arntl*, *Clock*, *Nr1d1*, *Per1*, *Cry1*, *Nfil3*) in a dose-dependent manner, involving either a ≥ 3.3-fold suppression in amplitude or complete loss of oscillation. Accordingly, protein levels (ARNTL, REV-ERBα, NFIL3) and genomic binding (ARNTL) of select regulators were reduced and arrhythmic following treatment. As a result, the oscillating expression of 99.6% of 5,636 clock-controlled hepatic genes was abolished including genes associated with the metabolism of lipids, glucose/glycogen, and heme. For example, TCDD flattened expression of the rate-limiting enzymes in both gluconeogenesis (*Pck1*) and glycogenesis (*Gys2*), consistent with the depletion and loss of rhythmicity in hepatic glycogen levels. Examination of polar hepatic extracts by untargeted mass spectrometry revealed that virtually all oscillating metabolites lost rhythmicity following treatment. Collectively, these results suggest TCDD disrupted circadian regulation of hepatic metabolism, altering metabolic efficiency and energy storage.

## Introduction

In response to the rotation of the Earth and the ensuing light-dark cycles, animals have evolved a ~24-hour (h) circadian clock that entrains physiological activities such as sleep and feeding to specific times of the day. Circadian oscillations enable cells to anticipate upcoming functional needs, imparting the organism with competitive advantages associated with fitness and survival including enhanced growth and longevity, improved reproductive success, and effective predator/prey relationships^1^. Maintaining rhythmicity of circadian behaviors is critical for optimal health, while disruptions such as shift work, jet lag, binge eating, and sleep restriction are associated with various pathogenic states in humans and rodent models including complex metabolic diseases (e.g. non-alcoholic fatty liver disease (NAFLD)), mental health disorders, cancer, and accelerated aging^2–6^.

The circadian system consists of a hierarchical network of tissue-specific peripheral clocks coordinated by a master pacemaker in the suprachiasmatic nucleus (SCN) of the hypothalamus. The SCN master clock is the only oscillator in the circadian network directly entrained by light detected at the retina^7^. In turn, this master clock generates oscillations in systemic cues (e.g. endocrine signals) and behavioral cycles (e.g. feeding/fasting, activity/rest) that synchronize cell-autonomous peripheral clocks to ensure phase coherence^8,9^. For example, the hepatic peripheral clock ensures that genes associated with nutrient transport, metabolism, and detoxification are coordinated with feeding/fasting cycles^3^. Temporal separation of incompatible or opposing metabolic processes also minimizes futile cycles while optimizing metabolic efficiency, energy use/storage, and cellular function. Approximately 40% of all genes in the mouse genome are circadian-regulated in at least one tissue, while the liver expresses the largest number of rhythmic genes (~11–16% of all detected transcripts), with ~50% of hepatic metabolites exhibiting oscillating levels^10–12^.

At the cellular level, circadian clocks comprise autonomous, interlocking transcription-translation feedback loops^13^. The positive limb involves the core activators circadian locomotor output cycles kaput (CLOCK), brain and muscle ARNT-like 1 (BMAL1, aka ARNTL), and neuronal PAS domain protein 2 (NPAS2, a CLOCK paralog), which are all members of the basic helix-loop-helix (bHLH) Per-Arnt-Sim (PAS) protein family. ARNTL forms a heterodimer with either CLOCK or NPAS2, which binds to E-box-containing response elements and induces cryptochrome (*Cry1*, *Cry2*) and period (*Per1*, *Per2*, *Per3*) transcription. Accumulating CRY and PER dimerize and interfere with ARNTL/CLOCK activity, thereby repressing their own transcription to create the negative limb of the loop. The ARNTL/CLOCK complex also induces the transcription of the *Nr1d1* (encodes REV-ERBα) and *Nr1d2* (encodes REV-ERBβ) repressors, which compete with the retinoic acid-related orphan receptor (ROR) α, β and γ activators for ROR-response elements (ROREs). REV-ERBα and β rhythmically repress *Arntl* transcription, creating a second feedback loop within the core clock. Several D-box-binding transcription factors are also regulated by ARNTL/CLOCK including D-site albumin promoter binding protein (DBP), thyrotroph embryonic factor (TEF), hepatic leukemia factor (HLF), and interleukin-3 regulated nuclear factor (NFIL3, aka E4BP4). Interactions between these loops generate transcriptional oscillation patterns in circadian-controlled target genes, where the expression phase is determined by the combination of E-boxes, ROREs, and D-boxes within each gene’s regulatory region.

The aryl hydrocarbon receptor (AhR) and its binding partner the aryl hydrocarbon receptor nuclear translocator (ARNT) are also members of the bHLH-PAS family and exhibit significant sequence homology with CLOCK and ARNTL, respectively^14–16^. Interestingly, several endogenous ligands of the AhR are photo-oxidation products of tryptophan (e.g. 6-formylindolo[3,2-b]carbazole (FICZ)), suggesting the AhR regulates biological rhythms in response to light-derived chemical messengers^17,18^. Additionally, AhR-deficient mice exhibit exacerbated behavioral responses to shifts in light cycles, increased amplitudes in core clock gene expression, and alterations in circadian-controlled metabolites (e.g. glucose, insulin, triglycerides), further demonstrating a role for AhR in circadian regulation^19^.

2,3,7,8-Tetrachlorodibenzo-*p*-dioxin (TCDD) is the prototypical ligand for a structurally diverse group of synthetic chemicals, natural products, and endogenous metabolites that activate the AhR^20^. Ligand binding causes the cytoplasmic AhR to dissociate from its chaperone proteins, followed by translocation to the nucleus and heterodimerization with ARNT. The ligand-bound AhR/ARNT complex then binds to dioxin response elements (DREs) within the promoter region of target genes, leading to recruitment of transcriptional co-regulators and differential gene expression^21^. Studies have also demonstrated differential gene expression following AhR binding within DNA regions lacking a DRE^22–25^. In mice, TCDD-elicited activation of the AhR affects the master clock, altering oscillations in circadian locomotor activity and expression patterns of core clock genes (*Arntl*, *Per1*) within the SCN^26,27^. Furthermore, AhR activation alters the rhythmic expression of core circadian regulators within the peripheral clocks of the liver, ovaries, and hematopoietic stem cells^28–30^.

Our previous studies detected TCDD-elicited differential expression of several core clock regulators and circadian-controlled target genes^31^. In addition to sigmoidal, exponential, and linear dose-response curves, a surprising number of genes also exhibited U- or inverted U-shaped curves, potentially reflecting rhythmic expression patterns^32,33^. TCDD also dysregulated porphyrin biosynthesis and increased hepatic levels of heme^31^, a cofactor for several core clock regulators including REV-ERBα/β, NPAS2, CLOCK, and PER2^34–37^. However, due to the large number of animals used to assess dose-response relationships, tissues were harvested over several hours, introducing confounding effects on circadian-regulated gene expression. The current study was designed to investigate the effects of TCDD on the diurnal rhythmicity of the hepatic transcriptome and metabolome, focusing on pathways associated with the progression of steatosis to steatohepatitis with fibrosis. We report TCDD abolished or dampened the rhythmicity of the core clock regulators at the mRNA, protein, and functional levels, collapsing the oscillation of virtually all circadian-controlled hepatic genes and metabolites. This loss of rhythmicity indicates optimal hepatic metabolism was decoupled from nutrient availability, compromising metabolic efficiency, energy storage, enterohepatic circulation, and redox homeostasis.

## Materials and Methods

### Animal treatment and sample collection

Postnatal day 25 (PND25) male C57BL/6 mice weighing within 10% of each other were obtained from Charles River Laboratories (Kingston, NY) and housed in Innovive Innocages (San Diego, CA) containing ALPHA-dri bedding (Shepherd Specialty Papers, Chicago, IL) in a 23 °C environment with 30–40% humidity. Mice were provided Aquavive water (Innovive) and Harlan Teklad 22/5 Rodent Diet 8940 (Madison, WI) *ad libitum*. Mice were entrained to a 12 h/12 h light/dark cycle for ~2 weeks prior to treatment and were maintained on this schedule throughout the treatment period. Mice (PND41) were orally gavaged at zeitgeber time (ZT) 0 with sesame oil vehicle (Sigma-Aldrich, St. Louis, MO) or 30 μg/kg body weight TCDD (AccuStandard, New Haven, CT) every 4 days (d) for a total of 7 exposures (Supplementary Fig. S1). The first gavage was administered on day 0 of the study and the final gavage was administered on day 24. Beginning at ZT0 on day 28, vehicle- and TCDD-treated mice were weighed and euthanized every 3 h ( ± 15 min) for 24 h (8 time points). Mice at the ZT0, 3, 6, and 9 time points were euthanized with the lights on, while mice at the ZT12, 15, 18, and 21 time points were euthanized under minimal light (overhead lights off, shielded from lamp light by a partial wall). The number of mice euthanized at each time point is listed in Supplementary Fig. S1. Differences in the number of mice within each group were due to animal losses (death or euthanasia prior to day 28) that can specifically be attributed to poor initial health (e.g. overgrown teeth, runts) and/or fighting/bullying. For animals exhibiting weight loss near the end of the study, treatment-related effects may have also been a factor. Livers were removed, weighed, flash frozen in liquid nitrogen, and stored at −80 °C. The relative liver weight (RLW) was calculated by dividing total liver weight by the terminal body weight. Hepatic TCDD levels resulting from this dosing regimen have previously been published^38^. The 30 μg/kg TCDD dose was chosen to compensate for the relatively short study duration compared to lifelong cumulative human exposure from diverse AhR ligands, the bioaccumulative nature of halogenated AhR ligands, and differences in TCDD’s metabolism and half-life (humans: 1–11 years^39,40^, mice: 8–12d^41,42^). Repeated dosing with 30 μg/kg TCDD elicits steatohepatitis with mild fibrosis and negligible necrosis/apoptosis in male mice^32^. Using this dosing regimen allowed us to examine circadian-regulated metabolic pathways which contribute to the progression of steatosis to steatohepatitis with fibrosis.

Food consumption was monitored in a separate cohort of male C57BL/6 mice exposed to the same treatment regimen of sesame oil vehicle or 30 µg/kg TCDD. Four cages of co-housed mice were included per treatment group. The food trough containing the rodent chow for each cage was weighed daily at ZT0. Food consumption was calculated based on the number of mice in the cage. For the dose-response analysis, a separate cohort of male C57BL/6 mice was orally gavaged at ZT0 with either sesame oil vehicle or 3, 10, or 30 μg/kg TCDD every 4 days for 7 exposures, as described above. Mice were euthanized on day 28 at either ZT0–3 or ZT5.5–8.5 and liver samples were flash frozen. A separate cohort of male C57BL/6 mice was orally gavaged at ZT2–4 with a single bolus dose of sesame oil vehicle or 30 µg/kg TCDD. Mice were euthanized 2 h after treatment (ZT4–6) and livers were collected for chromatin immunoprecipitation (ChIP) analysis. Dosing and euthanasia time points for the ChIP analysis were chosen to correspond with peak ARNTL binding (ZT6)^43^. All animal handling procedures were performed with the approval of the Michigan State University (MSU) Institutional Animal Care and Use Committee, in accordance with ethical guidelines and regulations.

### RNA-Seq analysis

Frozen liver samples were homogenized in TRIzol using a Mixer Mill 300 tissue homogenizer (Retsch, Germany) and total RNA was isolated as previously described^44^. RNA was quantified using a Nano-drop spectrophotometer (Thermo Scientific, Wilmington, DE) at 260 nm. Purity was assessed using the A_260_/A_280_ ratio and quality was analyzed using the Caliper LabChip GX (Perkin Elmer, Waltham, MA).

Hepatic gene expression at 3 h intervals was examined using RNA-Seq performed at the MSU Research Technology Support Facility (RTSF) Genomics Core (rtsf.natsci.msu.edu/genomics). Hepatic libraries from three individual mice (n = 3) were prepared using the Illumina TruSeq RNA Sample Preparation Kit (Illumina, San Diego, CA). Libraries were quantified and sequenced as previously described, at a read depth of ~30 M per sample^31,33^. Reads were assessed for quality using FASTQC v0.11.3 and then mapped to the mouse reference genome (GRCm38 release 81) using Bowtie2 v2.2.6 and TopHat2 v2.1.0. The RNA-Seq dataset was deposited in the Gene Expression Omnibus (GEO; accession number GSE119780). For TCDD-mediated differential gene expression, fold changes were calculated relative to vehicle controls at each ZT. Genes were considered differentially expressed if |fold change| ≥ 1.5 and statistical P1(t) value ≥ 0.8 at one or more time points.

### Quantitative Real-Time Polymerase Chain Reaction (qRT-PCR)

qRT-PCR was used to examine dose-dependent changes in hepatic gene expression. For each sample, cDNA was synthesized from 1 µg of total RNA using the High-Capacity cDNA Reverse Transcription Kit, as described by the manufacturer (Applied Biosystems, Foster City, CA). PCR amplification was conducted on a Bio-Rad CFX Connect Real-Time PCR Detection System using iQ SYBR Green Supermix, according to the manufacturer’s protocol (Bio-Rad, Hercules, CA). Gene expression was normalized to the geometric mean of two housekeeping genes (*Actb* and *Gapdh*), and fold changes relative to vehicle controls were calculated using the 2^−∆∆CT^ method. Forward and reverse primer sequences and amplicon sizes are provided in Supplementary Table S1.

### Protein quantification – capillary electrophoresis

Liver samples were homogenized in RIPA buffer supplemented with protease inhibitor cocktail (Sigma) using a Polytron PT2100 homogenizer (Kinematica, Lucerne, Switzerland) and sonicated on ice. Samples were centrifuged and total protein in the supernatant was measured using the bicinchoninic acid (BCA) assay (Sigma). The WES capillary electrophoresis system (ProteinSimple, San Jose, CA) was used with the following antibodies from Cell Signaling (Danvers, MA): ARNTL (#14020), REV-ERBα (#13418), and NFIL3 (#14312). Primary antibodies were detected using a goat anti-rabbit secondary antibody conjugated to horseradish peroxidase. Chemiluminescence signals were analyzed with Compass software (ProteinSimple). Target protein levels were normalized to total protein.

### ChIP and putative DRE identification

ChIP-PCR was used to: (i) investigate hepatic genomic ARNTL binding in male mice orally gavaged with sesame oil vehicle or 30 µg/kg TCDD every 4d for 28d, and (ii) compare genomic binding of AhR, ARNTL, and CLOCK in male mice 2 h after treatment with sesame oil vehicle or 30 µg/kg TCDD. Hepatic chromatin was prepared using the *tru*ChIP Tissue Chromatin Shearing Kit (Covaris, Woburn, MA) according to the manufacturer’s instructions with minor modifications. Briefly, frozen liver samples (~120 mg) were homogenized using a Polytron PT2100 homogenizer (Kinematica) in cold PBS. Protein-DNA complexes were cross-linked in 1% formaldehyde for 10 min and chromatin was sheared for 10 min in a 1 mL AFA Fiber milliTUBE using the M220 Focused-Ultrasonicator (Covaris). Size distributions of dsDNA fragments were assessed by running a DNA 1200 chip on the Agilent 2100 Bioanalyzer (Santa Clara, CA), to verify that ≥ 70% of fragments were within 150–700 bp. Triton X-100 (10%) was added to chromatin to achieve a final concentration of 1%. Cross-linked DNA was immunoprecipitated with rabbit antibodies as previously described^45^: IgG (#2729, Cell Signaling), ARNTL (#14020, Cell Signaling), CLOCK (ab3517, Abcam, Cambridge, MA), or AhR (BML-SA210, Enzo, Farmingdale, NY). ChIP DNA was purified using a QIAquick PCR purification kit (QIAGEN, Hilden, Germany) and eluted in 40 µL water. ChIP DNA and input DNA (diluted 100-fold) was quantified using qRT-PCR conducted on a Bio-Rad CFX Connect Real-Time PCR Detection System (Hercules, CA) as previously described^44^. Previously identified binding sites were targeted using the forward and reverse primer sequences listed in Supplementary Table S2^25,31,43^. A negative control region on chromosome 6 was identified using our group’s AhR^31^ and ARNTL (unpublished) ChIP-Seq datasets. Percent input was calculated using 100% * 2^((Ct Input − 6.644) − Ct IP)^.

Hepatic AhR ChIP-Seq was previously performed on samples from male C57BL/6 mice 2 h following a single oral dose of 30 µg/kg TCDD^31^. The full ChIP-Seq dataset for the male liver is available on GEO (GSE97634). Putative DREs (pDREs) were previously identified^46^. Briefly, the mouse genome (mm10 GRCm38 build) was computationally searched for the DRE core consensus sequence 5′-GCGTG-3′. Each identified core was extended by 7 bp upstream and downstream. The resulting 19 bp sequences were scored using a position weight matrix constructed from bona fide functional DREs. Matrix similarity scores (MSS) ≥ 0.856 were considered to be pDREs. For annotation at the gene level, pDRE locations were compared against the regulatory region (10 kb upstream of the transcription start site together with 5′- and 3′-untranslated regions) and coding sequence of each mouse gene obtained from the University of California Santa Cruz (UCSC) genome browser. UCSC genome browser tracks indicating pDRE locations within the mouse genome are available at http://dbzach.fst.msu.edu/index.php/supplementarydata.html. The raw bedGraph file for the mouse pDRE analysis is available on Harvard Dataverse^47^.

### Glucose and glycogen assay

Liver samples (~50 mg) were homogenized in 6% perchloric acid (250 µl) using a Polytron PT2100 homogenizer (Kinematica). To hydrolyze the glycogen, 25 µl of 1 M NaHCO_3_ and 125 µl of 2 mg/ml amyloglucosidase (Sigma-Aldrich) was added to 50 µl of the homogenate. Samples were incubated and shaken for 2 h at 37 °C and then centrifuged to remove debris. Glycogen and glucose were quantified using the glucose assay kit (Pointe Scientific, Canton, MI) with a M200 plate reader (Tecan, Durham, NC). Total hepatic glycogen levels were corrected using hepatic glucose levels and expressed as glucose units.

### Untargeted metabolomics analysis of hepatic extracts

Polar metabolites were extracted from liver samples using methanol:water:chloroform as previously described^48^. Briefly, flash frozen liver samples (~25 mg) were homogenized (Polytron PT2100, Kinematica) in a mixture of HPLC-grade methanol and water (5:3 ratio) containing all 20 ^13^C-,^15^N-labelled amino acids (Sigma; 767964) as internal standards. HPLC-grade chloroform (methanol:water:chloroform ratio 5:3:5) was added, vortexed, shaken for 15 min at 4 °C, and centrifuged at maximum speed (3000 × g) to achieve phase separation. The methanol:water phase containing the polar metabolites was transferred, dried under nitrogen gas at room temperature, and stored at −80 °C. Prior to analysis, the dried metabolite extracts were resuspended in 300 µL HPLC-grade water. For analysis in negative mode, 3 parts of the resuspended polar extract was diluted with 1 part 4X mobile phase (40 mM tributylamine + 60 mM acetic acid in 88/12 H_2_O/MeOH). For analysis in positive mode, 3 parts of the resuspended polar extract was diluted with 7 parts acetonitrile. Diluted samples were centrifuged at 15,000 g for 10 minutes to remove any remaining protein and the supernatant was transferred to autosampler vials.

Extracts were examined by untargeted liquid chromatography mass spectrometry (LCMS) using an Acquity UPLC System (Waters, Milford, MA) coupled with an Xevo G2-XS Quadrupole Time of Flight (QTof) mass spectrometer (Waters) run in MS^E^ continuum mode. For negative mode analysis, ion-pairing reverse phase chromatography was performed on an Ascentis Express column (C18, 5 cm × 2.1 mm, 2.7 µm, Sigma) using a modified version of a previously described method^48^ with the following LC parameters: injection volume, 10 µl; column temperature, 30 °C; and flow rate, 400 µl/min. The LC solvents were solvent A: 10 mM tributylamine and 15 mM acetic acid in 97:3 water:methanol (pH 4.95) and solvent B: methanol. Elution from the column was performed over 11 min with the following gradient: t = 0, 0% solvent B, flow rate 0.4 ml/min; t = 1, 0% solvent B, flow rate 0.4 ml/min; t = 2, 20% solvent B, flow rate 0.3 ml/min; t = 3, 20% solvent B, flow rate 0.25 ml/min; t = 5, 55% solvent B, flow rate 0.15 ml/min; t = 8, 95% solvent B, flow rate 0.15 ml/min; t = 8.5, 95% solvent B, flow rate 0.15 ml/min; t = 9, 0% solvent B, flow rate 0.4 ml/min; t = 11, 0% solvent B, flow rate 0.4 ml/min. Mass spectra were acquired using negative-mode electrospray ionization run in MS^E^ continuum mode. The capillary voltage was 2,500 V and cone voltage was 40 V. Nitrogen was used as cone gas and desolvation gas, with flow rates of 50 and 600 L/h, respectively. The source temperature was 100 °C, and desolvation temperature was 300 °C. Argon was used as collision gas.

For positive mode analysis, normal phase chromatography was performed using an Acquity UPLC Ethylene Bridged Hybrid (BEH) Amide column (10 cm × 2.1 mm, 2.7 µm, Waters) with the following LC parameters: injection volume, 10 µl; column temperature, 40 °C; and flow rate, 300 µl/min. The LC solvents were solvent A: 10 mM ammonium formate and 0.1% formic acid in water (pH 3.26) and solvent B: acetonitrile. Elution from the column was performed over 10 min with the following gradient: t = 0, 99% solvent B, flow rate 0.3 ml/min; t = 7, 50% solvent B, flow rate 0.3 ml/min; t = 8, 50% solvent B, flow rate 0.3 ml/min; t = 8.01, 99% solvent B, flow rate 0.3 ml/min; t = 10, 99% solvent B, flow rate 0.3 ml/min. Mass spectra were acquired using positive-mode electrospray ionization run in MS^E^ continuum mode. The capillary voltage was 3,000 V and cone voltage was 35 V. Nitrogen was used as cone gas and desolvation gas, with flow rates of 25 and 600 L/h, respectively. The source temperature was 100 °C, and desolvation temperature was 350 °C. Argon was used as collision gas. Progenesis QI (Waters) was used for peak processing. Metabolites were identified using the Human Metabolome Database (HMDB). Each sample was normalized to both liver weight and ^13^C-,^15^N-labelled amino acid standards: tryptophan and leucine for negative mode; phenylalanine and tyrosine for positive mode.

### Data analysis and data availability

The JTK_CYCLE package (v3) for R (v3.2.4) was used to assess rhythmicity of the transcriptomic and metabolomic datasets, as well as tissue weights, protein levels, and ChIP-PCR results^49^. Endpoints were considered to exhibit diurnal rhythmicity if BH q-value ≤ 0.1 for a period range of 21–24 h.

The Database for Annotation, Visualization, and Integrated Discovery (DAVID) v6.8 was used to identify enriched functional clusters within hepatic genes (i) exhibiting rhythmicity in controls but not TCDD-treated mice, and (ii) differentially expressed (|fold change| ≥ 1.5; P1(t) ≥ 0.8) by TCDD at three or more time points^50^. Only Gene Ontology (GO) Biological Processes were considered. The *mummichog* algorithm within MetaboAnalyst v4.0 (MS Peaks to Pathways module) was used to identify enriched KEGG pathways within mass spectrometry peaks that lost their rhythmicity following TCDD treatment^51^. Enrichment scores (ES; −log(p-value)) ≥ 1.3 were considered significant. Compound identifications determined by the *mummichog* analysis were used in select cases where the Progenesis QI software failed to identify a peak (e.g. heme).

The RNA-Seq analysis of hepatic gene expression at 3 h intervals is available in GEO (accession number GSE119780). Primer sequences for the qRT-PCR and ChIP-PCR analyses are listed in Supplementary Tables S1 and S2, respectively.

## Results

### TCDD abolished daily oscillations in relative liver weight

**Figure 1:**
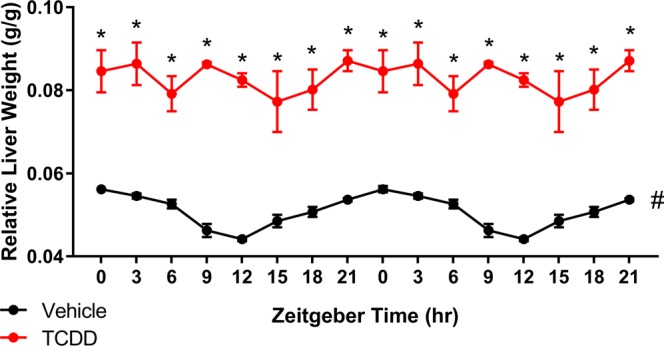
Relative liver weight (RLW) of male C57BL/6 mice orally gavaged with sesame oil vehicle or 30 µg/kg TCDD every 4 days for 28 days. Data points represent the average of at least 3 individual mice ± standard error of the mean. Statistical significance (*p ≤ 0.05) between vehicle and TCDD was determined using a two-way ANOVA analysis followed by Sidak’s multiple comparison test. JTK_CYCLE determined RLW in control animals exhibited daily oscillations (# BH q ≤ 0.1), while RLW rhythmicity was not detected in TCDD-treated mice. Data are double-plotted along the x-axis for better visualization of rhythmicity.

The effects of TCDD on body and liver weight were examined following oral gavage with sesame oil vehicle or 30 µg/kg TCDD every 4d for 28d. At euthanasia, the average body weight of treated mice was ~15% less than controls (Supplementary Fig. S2). JTK_CYCLE analysis determined that the RLW of controls oscillated in a diurnal manner, peaking at ZT0 (Fig. 1). This is consistent with previous mouse and rat studies in which both RLW and hepatocyte size were found to exhibit circadian oscillations over a 24 h period, reaching maximal size and weight at the end of the feeding phase (ZT0)^52,53^. TCDD increased RLW at each time point with a maximum increase of 1.9-fold at ZT12, comparable to RLW increases reported in previous studies^31,38^. Notably, the daily rhythmicity in RLW was lost following TCDD treatment (Fig. 1). TCDD had no effect on daily food consumption over the 28d treatment regimen (Supplementary Fig. S3), consistent with previous studies^54^. Therefore, TCDD-elicited alterations in body weight, RLW, and hepatic rhythmicity are not driven by changes in overall food intake.

### Loss of hepatic rhythmic gene expression following AhR activation

**Figure 2:**
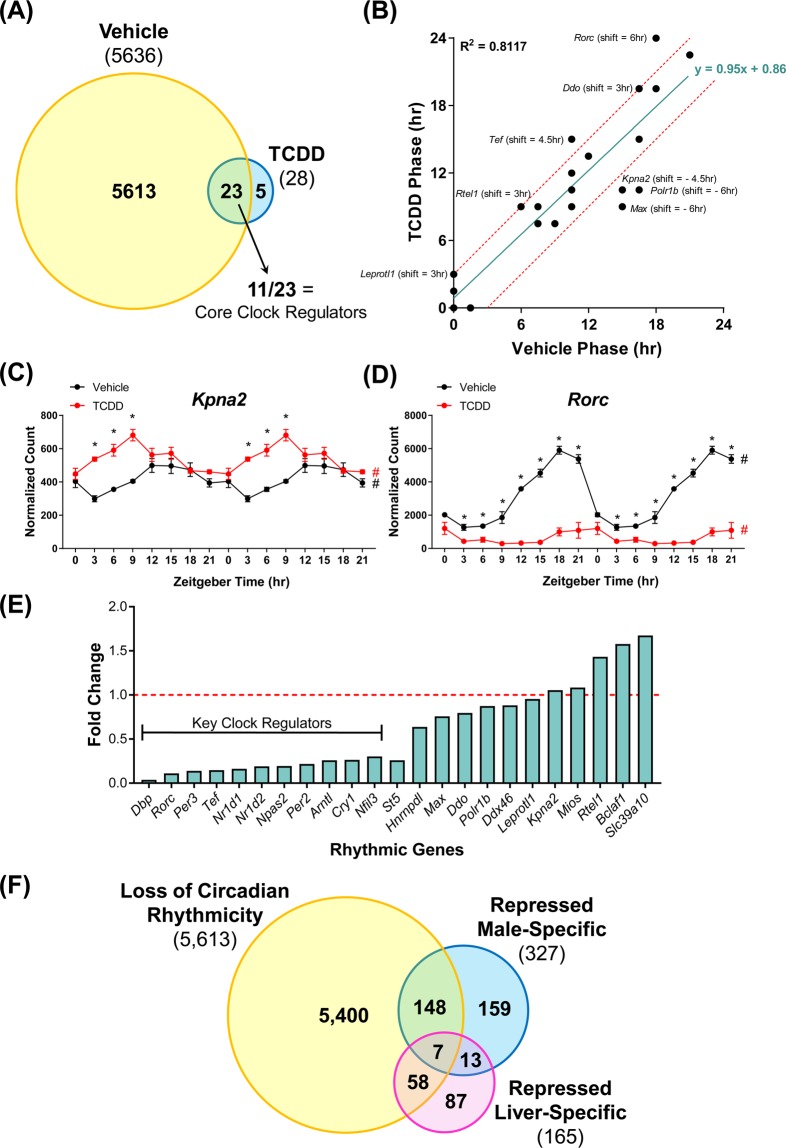
The effect of TCDD on the diurnal rhythmicity of hepatic gene expression in male C57BL/6 mice orally gavaged with sesame oil vehicle or 30 μg/kg TCDD every 4 days for 28 days. (**A**) The number of hepatic genes which exhibited rhythmic expression in vehicle- and TCDD-treated mice. 5,613 of 5,636 (99.6%) rhythmic genes lost their diurnal oscillation pattern following treatment, while 23 genes exhibited rhythmicity in both vehicle- and TCDD-treated mice. Rhythmicity was determined using JTK_CYCLE (BH q ≤ 0.1). (**B**) Correlation between the acrophases (time at which cycle peaks/crests) of rhythmic expression in vehicle- and TCDD-treated mice. The linear regression line is shown in green, while the red dashed lines represent acrophase shifts of ± 3 h. Genes falling within the red dashed lines exhibited negligible acrophase shifts of ≤ 1.5 h. TCDD decreased the acrophase of (**C**) *Kpna2* (as well as *Polr1b* and *Max*, not shown), while increasing the acrophase of (**D**) *Rorc* (as well as *Leprotl1*, *Rtel1*, *Tef*, and *Ddo*, not shown). Data points represent the average of 3 individual mice ± standard error of the mean. Posterior probabilities (*P1(t) ≥ 0.80) comparing vehicle and TCDD were determined using an empirical Bayes method. Diurnal rhythmicity was assessed using JTK_CYCLE (# BH q ≤ 0.1). Data in (**C**,**D**) are double-plotted along the x-axis for better visualization of rhythmicity. (**E**) Altered amplitudes (fold change) of hepatic genes exhibiting rhythmicity in both vehicle- and TCDD-treated mice. The red dashed line represents a fold change of 1 (i.e. treatment had no effect on amplitude). (**F**) Genes which lost rhythmicity were compared to previously identified sets of male-specific and liver-specific genes repressed by TCDD^56^.

Using RNA-Seq, TCDD-elicited hepatic transcriptomic changes were assessed at 3 h intervals over a 24 h period. JTK_CYCLE analysis of the RNA-Seq data detected 5,636 hepatic genes exhibiting diurnal rhythmic expression (BH q-value ≤ 0.1; period = 21–24 h) in control mice (Fig. 2A), equivalent to 25.7% of the 21,896 genes expressed in the liver. This is higher than the ~11 to 16% of hepatic genes reported to exhibit circadian oscillations in other studies^10,11^, but less than a recent 37% estimate^12^. These discrepancies are likely due to differences in the BH q-value cut-off, transcriptomic platforms, sampling intervals, and statistical power. Specifically, 15 core hepatic clock regulators exhibited oscillating expression including (i) the E-box binding transcription factors *Arntl* (aka *Bmal1*), *Clock*, and *Npas2*, (ii) the PER/CRY genes *Per1*, *Per2*, *Per3*, *Cry1*, and *Cry2*, (iii) the RORE-binding transcription factors *Nr1d1* (encodes REV-ERBα), *Nr1d2* (encodes REV-ERBβ), and *Rorc* (encodes RORϒ), and (iv) the D-box binding transcription factors *Dbp*, *Tef*, *Hlf*, and *Nfil3*. Hepatic expression of the RORE-binding transcription factor *Rora* did not exhibit rhythmicity, consistent with previous reports that it lacks circadian oscillation in peripheral tissues^55^. Collectively, these results confirmed that our study was appropriately designed to examine the effects of persistent AhR activation on the hepatic circadian clock.

The classical AhR target genes *Cyp1a1*, *Cyp1a2*, and *Cyp1b1* did not exhibit rhythmicity in vehicle mice according to JTK_CYCLE analysis. In TCDD-treated mice, these cytochrome P450s were induced 542.9-, 19.5-, and 562.7-fold, respectively, confirming hepatic AhR activation. Overall, 8,970 arrhythmic genes were differentially expressed by TCDD at one or more time points. According to JTK_CYCLE analysis, TCDD abolished the rhythmicity of 5,613 hepatic genes, equivalent to 99.6% of the genes which exhibited circadian regulation in controls. The coefficient of variation (COV = standard deviation / average) of the normalized log_2_-transformed RNA-Seq read counts (n = 3) for each circadian-regulated gene was calculated at each ZT, averaged across the 8 timepoints, and compared between vehicle and TCDD-treated mice (Supplementary Fig. S4). Linear regression analysis revealed a slope of 0.83, suggesting variation in circadian-regulated gene expression was roughly equivalent between control and treated mice.

Following TCDD treatment, only 23 circadian-regulated genes retained rhythmicity, while 5 acquired rhythmic expression (*Sdhaf2*, *Nsrp1*, *Marveld3*, *Folr1*, and *Gm17068*) (Fig. 2A). Linear regression analysis of the time at which each gene expression cycle peaks/crests (acrophase) between vehicle- and TCDD-treated animals revealed a slope of 0.95, suggesting TCDD had little effect on the acrophase of most genes. For example, of the 23 genes which maintained rhythmicity, 15 exhibited similar acrophases (±1.5 h) between control and TCDD-treated animals (Fig. 2B; Supplementary Table S3). TCDD increased the acrophase of *Rorc*, *Tef*, *Leprotl1*, *Rtel1*, and *Ddo* ≥ 3 h (+ve phase shift), and decreased the acrophase of *Kpna2*, *Max*, and *Polr1b* ≥ 4.5 h (−ve phase shift) (Fig. 2C,D). In contrast to limited effects on acrophase, TCDD altered the amplitude of most rhythmic genes. Of the 23 genes identified as rhythmic in both controls and treated mice, 15 exhibited a ≥ 1.3-fold reduction in amplitude, while only 3 genes increased in amplitude (*Rtel1*, *Bclaf1*, *Slc39a10;* 1.4-, 1.6-, 1.7-fold) (Fig. 2E). Overall, TCDD abolished the rhythmicity of the vast majority of clock-controlled hepatic genes, while those that continued oscillating following treatment exhibited reduced amplitudes (in most cases). Beyond the collapse of the hepatic circadian clock, previous studies have shown TCDD also causes the loss of liver-specific and sexually dimorphic gene expression^56^. Comparison of these gene sets revealed 47% of repressed male-specific genes and 39% of repressed liver-specific genes exhibited rhythmicity in vehicle- but not TCDD-treated mice (Fig. 2F).

### TCDD dampened rhythmicity of hepatic core clock regulators

**Figure 3:**
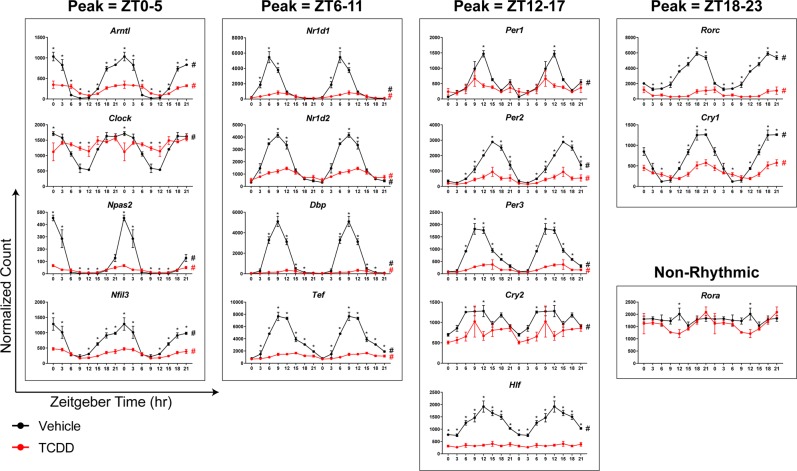
RNA-Seq analysis of core hepatic clock regulators in male C57BL/6 mice orally gavaged with sesame oil vehicle or 30 µg/kg TCDD every 4 days for 28 days. Data points represent the average of 3 individual mice ± standard error of the mean. Posterior probabilities (*P1(t) ≥ 0.80) comparing vehicle and TCDD were determined using an empirical Bayes method. Diurnal rhythmicity was assessed using JTK_CYCLE (# BH q ≤ 0.1). Genes are grouped by their acrophase (time at which gene expression peaks/crests in cycle) in vehicle mice. Data are double-plotted along the x-axis for better visualization of rhythmicity.

**Figure 4:**
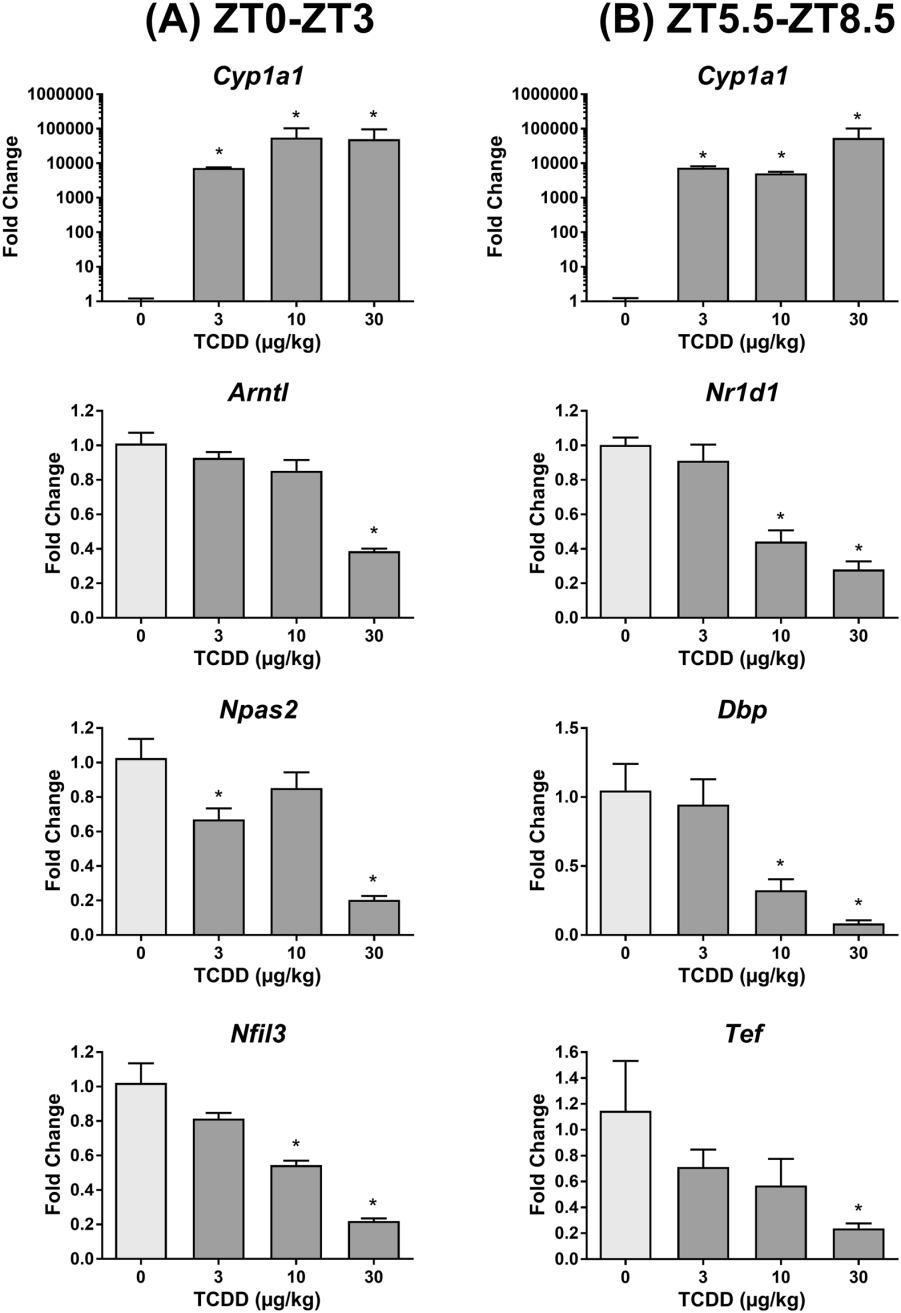
Dose-dependent effects of TCDD on the hepatic expression of core clock genes determined by quantitative real-time polymerase chain reaction (qRT-PCR). Male C57BL/6 mice were orally gavaged with sesame oil vehicle or 3–30 µg/kg TCDD every 4 days for 28 days. On day 28, livers were collected at either (**A**) ZT0–3 or (**B**) ZT5.5–8.5. Bars represent the average of at least 3 individual mice + standard error of the mean. Target genes were normalized to *Gapdh* and *Actb* expression. Statistical significance compared to vehicle controls (*p ≤ 0.05) was determined using a one-way ANOVA analysis followed by Dunnett’s post-hoc test.

The striking global loss in hepatic gene expression rhythmicity is likely due to the dampened expression of core circadian clock regulators. Notably, TCDD repressed hepatic expression of all 16 core clock regulators at one or more time points (Fig. 3; Table 1). Furthermore, the rhythmic expression of all core clock regulators was diminished by TCDD, involving either a ≥ 3.3-fold reduction in amplitude (*Arntl*, *Npas2*, *Nr1d1*, *Nr1d2*, *Rorc*, *Per2*, *Per3*, *Cry1*, *Nfil3*, *Dbp*, and *Tef*) or a complete loss of oscillation (*Clock*, *Per1*, *Cry2*, and *Hlf*). For example, the amplitude of *Dbp*, *Rorc*, and *Per3* was repressed 27.3-, 9.1-, and 7.2-fold by TCDD (Fig. 3; Table 1). qRT-PCR analysis confirmed that TCDD dose-dependently repressed select hepatic core clock genes (Fig. 4).

**Table 1:** Putative dioxin response elements (pDREs) and AhR binding within core clock genes.

Gene	Number of pDREs^a^	Number of AhR Binding Peaks^b^	Max AhR Enrichment Fold Change (AhR vs. IgG)	AhR Enrichment at a pDRE?	Rhythmic^c^ in VEH?	Rhythmic^c^ after TCDD?	Phase Shift (h) (TCDD - VEH)	Amplitude Fold Change (TCDD vs. VEH)
*Arntl*	2	4	2.2	No	Yes	Yes	1.5	−3.9
*Clock*	10	2	1.9	Yes	Yes	No	N/A	N/A
*Npas2*	14	2	2.3	No	Yes	Yes	0	−5.2
*Nr1d1*	1	2	3.2	Yes	Yes	Yes	1.5	−6.1
*Nr1d2*	0	3	2.2	No	Yes	Yes	1.5	−5.3
*Rora*	9	20	6.5	No	No	No	N/A	N/A
*Rorc*	3	2	2.9	No	Yes	Yes	6	−9.1
*Per1*	2	5	2.6	Yes	Yes	No	N/A	N/A
*Per2*	4	7	14.1	Yes	Yes	Yes	−1.5	−4.6
*Per3*	7	0	N/A	No	Yes	Yes	1.5	−7.2
*Cry1*	4	1	2.2	No	Yes	Yes	1.5	−3.8
*Cry2*	13	4	2.9	Yes	Yes	No	N/A	N/A
*Nfil3*	1	5	3.3	No	Yes	Yes	0	−3.3
*Dbp*	3	1	1.9	No	Yes	Yes	0	−27.3
*Tef*	5	2	2.0	Yes	Yes	Yes	4.5	−6.8
*Hlf*	2	9	7.9	Yes	Yes	No	N/A	N/A

^a^Putative dioxin response elements (pDREs; MSS ≥ 0.856) were identified by computationally querying the mouse genome^46^.

^b^AhR genomic enrichment was determined through ChIP-Seq analysis of male livers 2 h after treatment with 30 μg/kg TCDD^31^.

^c^Rhythmicity was determined using JTK_CYCLE (BH q-value ≤ 0.1; period = 21–24 h)^49^.

**Figure 5:**
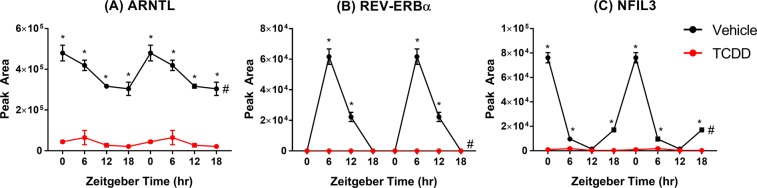
Hepatic protein levels of (**A**) ARNTL, (**B**) REV-ERBα, and (**C**) NFIL3 in male C57BL/6 mice orally gavaged with sesame oil vehicle or 30 µg/kg TCDD every 4 days for 28 days. Data points represent the average of 3 individual mice ± standard error of the mean measured using capillary electrophoresis (WES ProteinSimple System). Statistical significance (*p ≤ 0.05) between vehicle and TCDD was determined using a two-way ANOVA analysis followed by Sidak’s multiple comparison test. Diurnal rhythmicity was assessed using JTK_CYCLE (# BH q ≤ 0.1). Data are double-plotted along the x-axis for better visualization of rhythmicity.

Capillary electrophoresis (WES ProteinSimple System) was used to evaluate the effect of TCDD on hepatic protein levels of select clock regulators. JTK_CYCLE analysis (BH q-value ≤ 0.1; period = 21–24 h) confirmed ARNTL, REV-ERBα, and NFIL3 protein levels exhibited rhythmic oscillations in control samples. TCDD reduced hepatic ARNTL and NFIL3 protein levels 14.4- and 80.6-fold, respectively, while REV-ERBα protein was undetected in TCDD-treated mouse liver samples at every time point (Fig. 5). Furthermore, TCDD abolished the diurnal rhythmicity of these three proteins, consistent with reduced amplitudes in gene expression.

**Figure 6:**
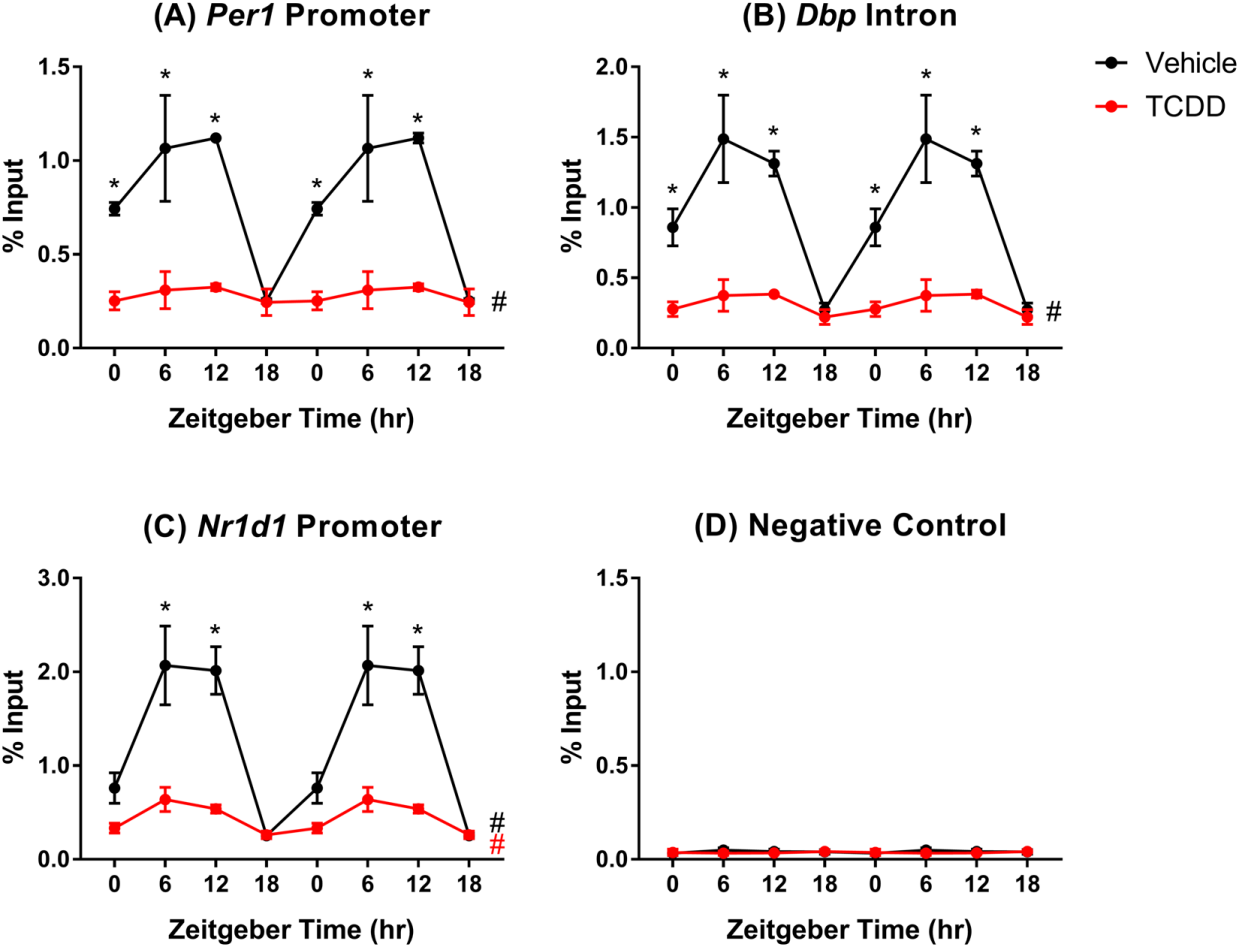
ARNTL genomic enrichment within target genes assessed using chromatin immunoprecipitation (ChIP). Liver samples were collected from male C57BL/6 mice following oral gavage with sesame oil vehicle or 30 µg/kg TCDD every 4 days for 28 days. Genomic enrichment for ARNTL was quantified by qRT-PCR using primers targeting previously identified ARNTL binding sites within (**A**) the *Per1* promoter (site 2), (**B**) a *Dbp* intron, and (**C**) the *Nr1d1* promoter as described in Supplementary Table S2. (**D**) A negative control region on chromosome 6 was used to confirm the specificity of the ANRTL immunoprecipitation. Data points represent the average % input of 3 individual mice ± standard error of the mean. Statistical significance (*p ≤ 0.05) between vehicle- and TCDD-treated mice was determined using a two-way ANOVA analysis followed by Sidak’s multiple comparison test. Diurnal rhythmicity was assessed using JTK_CYCLE (# BH q ≤ 0.1). Data are double-plotted along the x-axis for better visualization of rhythmicity.

ChIP-PCR was used to evaluate hepatic ARNTL genomic enrichment following persistent AhR activation over 28d. In control livers, oscillating genomic ARNTL binding within previously identified target genes was confirmed, with peak binding detected at ZT9. TCDD reduced ARNTL binding within *Per1*, *Dbp*, and *Nr1d1* 3.4-, 4.0-, and 3.7-fold, respectively. Moreover, ARNTL binding rhythmicity was abolished within *Per1* and *Dbp*, while the amplitude of rhythmicity at *Nr1d1* was reduced 5.9-fold (Fig. 6A–C). The specificity of the ARNTL immunoprecipitation was confirmed using a negative control region on chromosome 6 (Fig. 6D). These results demonstrate that TCDD dampened *Arntl* rhythmic mRNA expression, reduced ARNTL protein levels, and impaired ARNTL genomic binding within target genes.

Although rhythmic expression of most hepatic genes is regulated by the local molecular clock, some genes retain rhythmicity in the absence of a functional hepatic oscillator^57,58^. These “system-driven genes” are regulated by systemic oscillating cues including feeding/fasting cycles, body temperature fluctuations, and diurnal hormones (e.g. glucocorticoids). JTK_CYCLE analysis classified these system-driven genes as arrhythmic following TCDD treatment, with the exception of *Per2*. However, visual assessment suggests some of these genes still exhibit a diurnal oscillating trend (e.g. *Hsph1*, *Hspa1b*. *Hspa8*, *Hsp90aa1*, *Chordc1*, *Stip1*, *Fus*), albeit with reduced amplitude (Supplementary Figs S5 and S6). Despite discrepancies between visual assessment and JTK_CYCLE analysis, the oscillating expression of system-driven genes was altered by TCDD. In many cases, hepatic ChIP-Seq analysis identified increased AhR genomic binding in these genes^31^, implying disruption of their rhythmic expression may be a direct consequence of TCDD.

### Increased genomic AhR binding within core clock genes

A previously published hepatic RNA-Seq time-course analysis revealed differential expression of several E-box-containing core clock genes as early as 4 h after TCDD treatment, prior to any phenotypic effects, suggesting direct AhR-dependent regulation of the hepatic clock. Specifically, TCDD repressed *Per1* (2.0-fold; not-significant), *Per3* (3.0-fold), *Dbp* (3.3-fold), and *Tef* (1.8-fold) at 4 h, while *Per2* was induced 3.0-fold^59^. At least one pDRE (MSS ≥ 0.856) is present within the regulatory region (10 kb upstream of TSS to TES) of each mouse core clock gene with the exception of *Nr1d2*^46^. Furthermore, AhR ChIP-Seq analysis of male livers 2 h following TCDD treatment showed increased AhR genomic binding (fold change ≥ 1.9 vs. IgG) within the loci of all core clock genes except *Per3*^31^, providing strong evidence that hepatic core clock genes are direct AhR targets (Table 1). Among these 15 core clock genes, only 7 exhibited AhR enrichment at a site containing a pDRE, while the other 8 genes exhibited AhR enrichment within regions lacking a pDRE (Table 1). Therefore, TCDD-elicited disruption of hepatic rhythmicity likely involves both DRE-dependent and DRE-independent AhR signaling.

**Figure 7:**
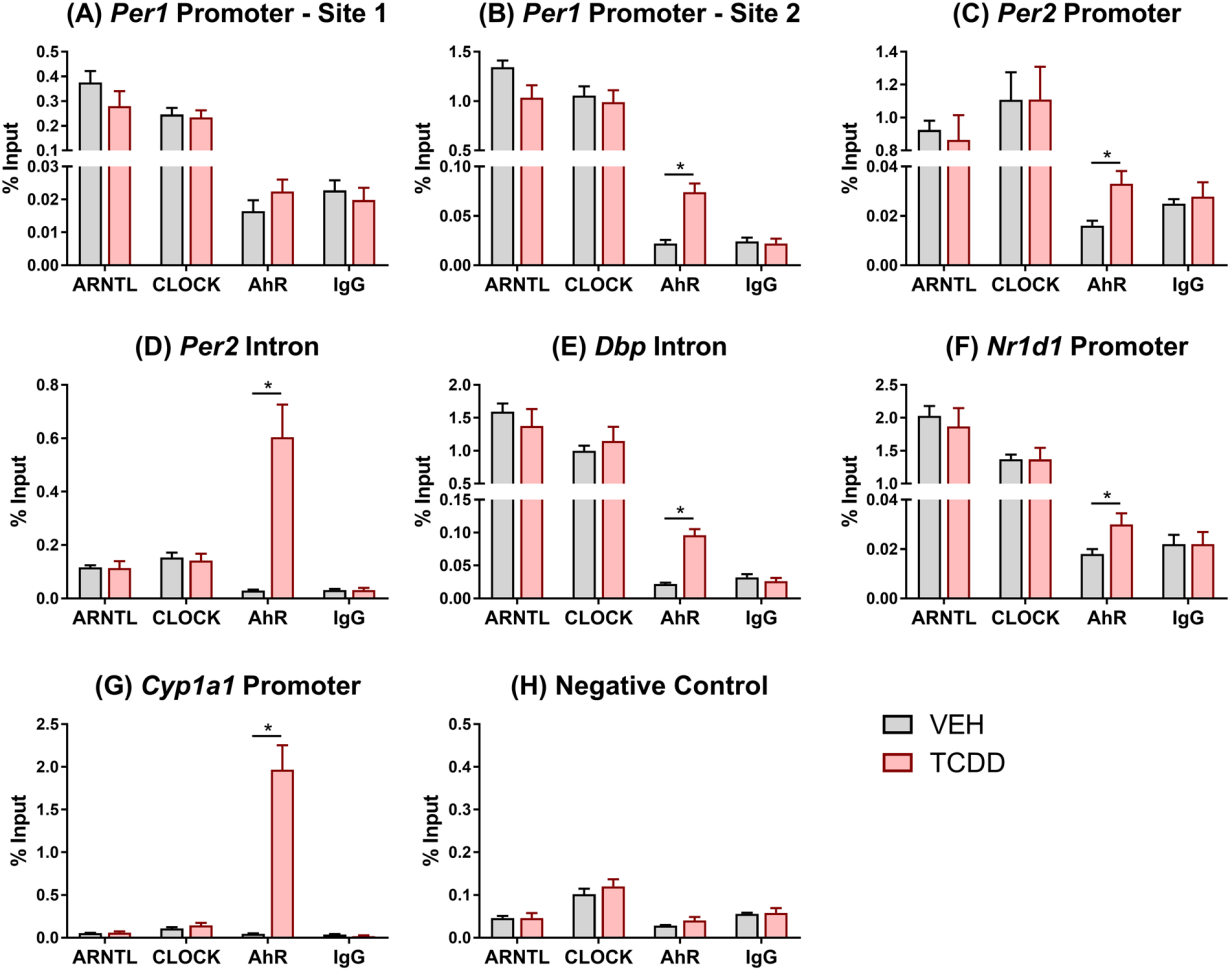
Comparison of ARNTL, CLOCK, and AhR genomic enrichment within target genes assessed using chromatin immunoprecipitation (ChIP). Liver samples were collected at ZT4–6 from male C57BL/6 mice 2 h following a single bolus dose of sesame oil vehicle or 30 µg/kg TCDD. Genomic enrichment was quantified by qRT-PCR using primers targeting regions within (**A**,**B**) *Per1*, (**C**,**D**) *Per2*, (**E**) *Dbp*, and (**F**) *Nr1d1* as described in Supplementary Table S2. (**G**) *Cyp1a1* was used as a positive control for AhR binding, while (**H**) a negative control region on chromosome 6 was used to confirm the specificity of each immunoprecipitation. Data points represent the average % input of 4–5 individual mice ± standard error of the mean. Statistical significance (*p ≤ 0.05) between vehicle- and TCDD-treated mice was determined using a t-test.

*In vitro* studies using Hepa1c1c7 cells treated with β-naphthoflavone (βNF) for 1.5 h report AhR interacts with ARNTL and binds at E-box response elements within the *Per1* promoter. This decreases ARNTL/CLOCK heterodimerization at E-boxes within *Per1* and represses its expression^25^. To further investigate DRE-independent mechanisms involved in circadian dysregulation *in vivo*, ChIP-PCR was used to compare AhR, ARNTL, and CLOCK genomic binding in liver samples of male mice orally gavaged with TCDD for 2 h. The primers described by Xu *et al*. (2010) were used to assess binding within the *Per1* promoter at a site containing a canonical E-box but no pDRE (promoter site 1)^25^. In contrast to *in vitro* βNF treatment, *in vivo* TCDD treatment had no effect on AhR, CLOCK, or ARNTL enrichment within this region (Fig. 7A). A 3.4-fold increase in AhR binding was detected further upstream of the *Per1* transcription start site (promoter site 2), near an E-box and pDRE. However, neither CLOCK nor ARNTL binding were affected by TCDD (Fig. 7B). Similarly, 2.1-fold enrichment in AhR binding was detected in a region containing a non-canonical E-box (5′-CACGTT-3′^60^) within the *Per2* promoter, despite a lack of pDREs. Again, AhR binding did not interfere with CLOCK or ARNTL binding at this site (Fig. 7C). AhR binding was also enriched 21.8-fold at a pDRE within a *Per2* intron. Despite a lack of canonical E-boxes, CLOCK and ARNTL binding were detected but unaffected by TCDD (Fig. 7D). AhR enrichment was also increased 4.3- and 1.8-fold within a *Dbp* intron and the *Nr1d1* promoter, respectively, with no effect on CLOCK or ARNTL binding (Fig. 7E,F). A 43.8-fold increase in AhR binding at a pDRE within the *Cyp1a1* promoter served as a positive control (Fig. 7G), while no enrichment was detected in the negative control region on chromosome 6 (Fig. 7H). Overall, TCDD-activated AhR did not interfere with ARNTL or CLOCK binding at the regions examined in this study.

### TCDD disrupts circadian regulation of hepatic metabolism

**Figure 8:**
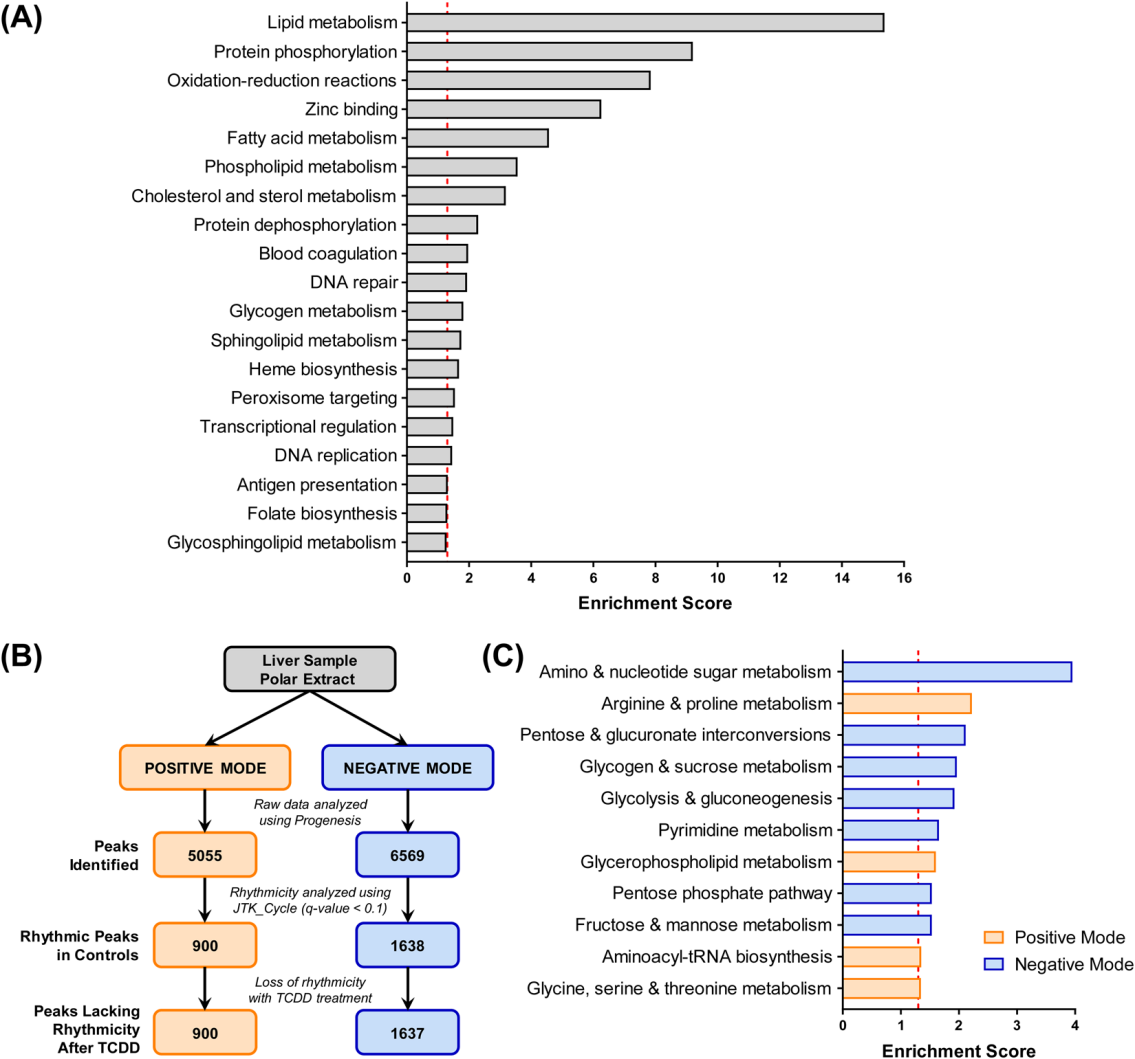
Functional analysis of hepatic genes and metabolites which lost their rhythmicity following TCDD treatment. Transcriptomic and metabolomic changes were assessed in male C57BL/6 mice orally gavaged with sesame oil vehicle or 30 µg/kg TCDD every 4 days for 28 days. (**A**) The Database for Annotation, Visualization, and Integrated Discovery (DAVID) v6.8 was used to identify enriched functional clusters within 2,804 hepatic genes with abolished rhythmicity and TCDD-elicited differential expression at three or more time points. (**B**) Flow chart summarizing the analysis of an untargeted metabolomics assessment of polar hepatic extracts run in both positive and negative mode. (**C**) The *mummichog* algorithm in MetaboAnalyst v4.0 (MS Peaks to Pathways) was used to identify enriched KEGG pathways in the 900 and 1,637 oscillating peaks identified in positive (orange) and negative (blue) mode, respectively, which lost their rhythmicity following TCDD treatment. Scores ≥1.3 (red dotted line) were considered significantly enriched.

To identify circadian-controlled biological processes and metabolic pathways affected by TCDD, a functional enrichment analysis was performed on 2,804 hepatic genes that: (i) exhibited rhythmicity in controls but not TCDD-treated mice, and (ii) were differentially expressed (|fold change| ≥ 1.5; P1(t) ≥ 0.8) by TCDD at three or more time points. DAVID identified 19 enriched functional clusters (ES ≥ 1.3) including metabolism of lipids (e.g. fatty acids, phospholipids, cholesterol/sterols, and sphingolipids), glycogen, and heme, as well as oxidation-reduction reactions and DNA repair (Fig. 8A). Indeed, the hepatic peripheral clock is known to regulate nutrient metabolism, oxidative defense, and DNA repair, facilitating synchronization with feeding/fasting cycles and ultraviolet (UV) radiation exposure during daylight^3^.

The effect of TCDD on circadian-controlled hepatic metabolism was further evaluated through an untargeted metabolomics analysis of the liver. Negative mode electrospray ionization of polar hepatic extracts detected 6,569 metabolite peaks, of which 1,638 (24.9%) were classified as rhythmic by JTK_CYCLE (BH q-value ≤ 0.1; period = 21–24 h). Similarly, the positive mode analysis detected 5,055 metabolite peaks, where 900 (17.8%) were rhythmic in controls (Fig. 8B). This fraction of rhythmic hepatic metabolites is comparable to the percentage of oscillating hepatic genes detected (25.7%), but lower than the ~50% reported in a targeted metabolomics study^12^. Following TCDD treatment, 1,637 of the 1,638 oscillating peaks detected in negative mode lost rhythmicity (99.9%), while 100% of the positive mode peaks were arrhythmic (Fig. 8B). Using the *mummichog* algorithm within MetaboAnalyst^51^, 11 enriched KEGG pathways were identified among the peaks which lost rhythmicity (7 in negative mode, 4 in positive mode), including several biological processes also enriched at the gene expression level (Fig. 8C). Glucose and glycogen metabolism, heme biosynthesis, bile acid homeostasis (Supplementary Information), and redox homeostasis (Supplementary Information) were examined further through the integration of transcriptomic, metabolomic, and enzymatic analyses.

### Glucose and glycogen metabolism

**Figure 9:**
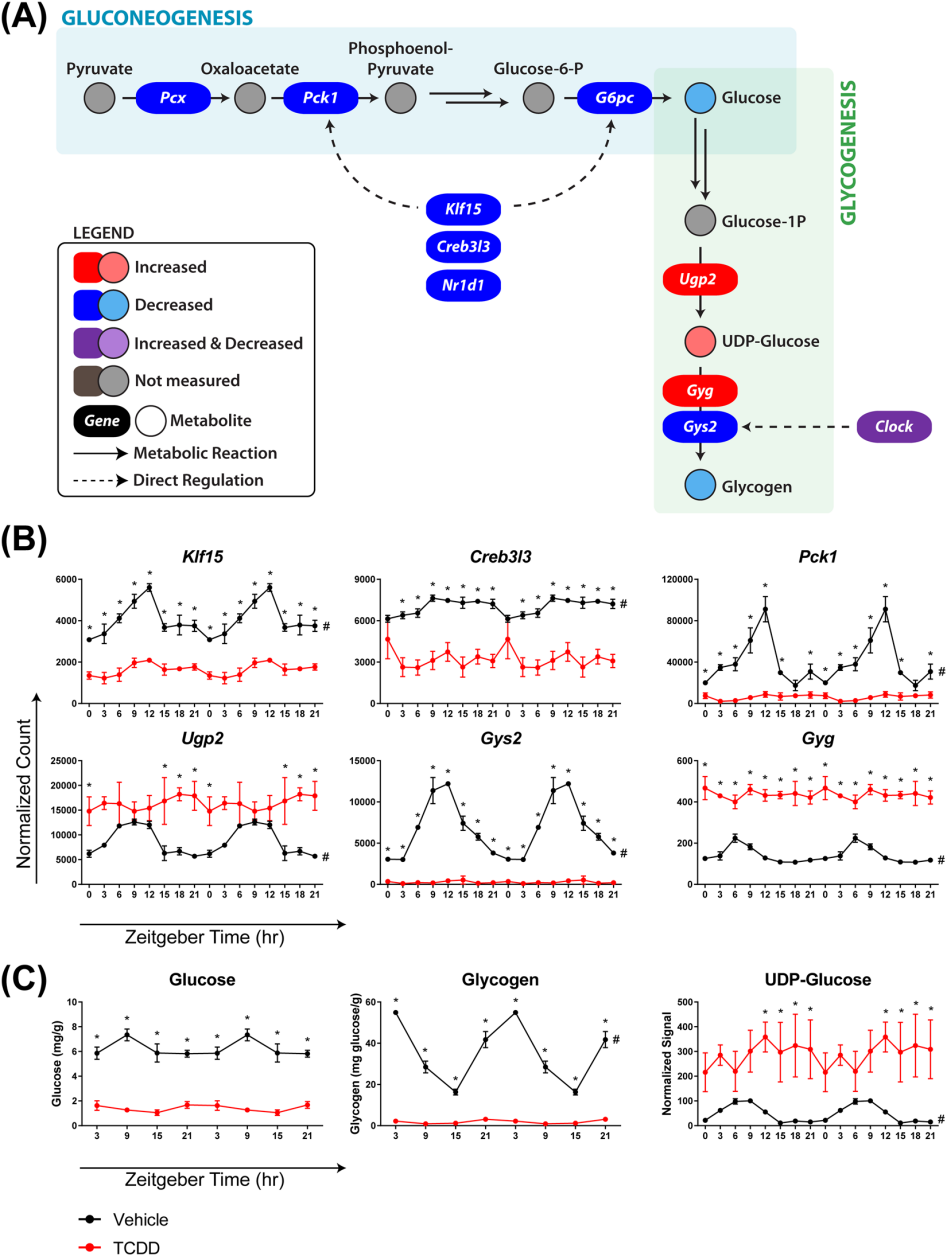
TCDD disrupts the circadian regulation of glucose homeostasis. Male C57BL/6 mice were orally gavaged with sesame oil vehicle or 30 µg/kg TCDD every 4 days for 28 days. (**A**) The effect of TCDD on hepatic gluconeogenesis and glycogenesis. TCDD-elicited changes in hepatic (**B**) genes and (**C**) metabolites involved in gluconeogenesis and glycogenesis. For genes, data points represent the average of 3 individual mice ± standard error of the mean (SEM), where posterior probabilities (*P1(t) ≥ 0.80) comparing vehicle and TCDD were determined using an empirical Bayes method. For metabolites, data points represent the average of 3–5 individual mice ± SEM, where statistical significance (*p ≤ 0.05) between vehicle and TCDD was determined using a 2-way ANOVA analysis followed by Sidak’s multiple comparison test. Diurnal rhythmicity was assessed using JTK_CYCLE (# BH q ≤ 0.1). Data are double-plotted along the x-axis for better visualization of rhythmicity.

During the active phase, glucose is primarily obtained through the consumption of dietary polysaccharides, while the breakdown of glycogen (glycogenolysis) and *de novo* glucose biosynthesis (gluconeogenesis) provide glucose during the fasting phase. Several key gluconeogenesis genes including phosphoenolpyruvate carboxykinase 1 (*Pck1*) and glucose-6-phosphatase (*G6pc*) are directly regulated by core clock transcription factors (e.g. REV-ERBα), as well as the circadian-regulated transcriptional activators Krüppel-like factor 15 (KLF15) and cAMP responsive element binding protein 3-like 3 (CREB3L3)^61–63^. Both *Klf15* and *Creb3l3* lost rhythmicity and were repressed (3.1- and 3.0-fold, respectively) by TCDD. Accordingly, *Pck1* and *G6pc* were repressed 17.7- and 6.9-fold, respectively, consistent with results reported in KLF15 and CREB3L3 knockout models^61,63^. *Pck1* rhythmic oscillation was concurrently abolished, while *G6pc* was classified as arrhythmic in both control and treated animals (Fig. 9). KLF15 also controls the availability of gluconeogenic precursors (e.g. pyruvate) by regulating amino acid catabolism and nitrogenous waste excretion (i.e. urea cycle). Several other KLF15 target genes were also repressed including glutamic pyruvic transaminase (*Gpt*; 12.3-fold), 4-hydroxyphenylpyruvic acid dioxygenase (*Hpd;* 3.7-fold), proline dehydrogenase (*Prodh;* 5.4-fold), tryptophan 2,3-dioxygenase (*Tdo2;* 2.0-fold), and ornithine transcarbamylase (*Otc;* 57.9-fold), impairing amino acid catabolism and gluconeogenic precursor availability. Consistent with this repression of key gluconeogenesis enzymes and regulators, hepatic glucose levels were reduced (up to 5.8-fold) at each time point (Fig. 9).

Excess glucose can be stored as glycogen and called upon as an energy source during fasting. As such, hepatic glycogen levels oscillate in a circadian manner peaking at the end of the active phase (ZT0) (Fig. 9). TCDD not only decreased glycogen levels (up to 31.4-fold), but also abolished the diurnal rhythmic pattern. This is consistent with the 88.7-fold repression and loss of rhythmicity in glycogen synthase 2 (Gys2) (Fig. 9), the rate-limiting step of hepatic glycogenesis which is directly regulated by CLOCK^64^. Loss of circadian regulation of hepatic *Gys2* expression and glycogen content was also reported in CLOCK mutant mice^64^. Paradoxically, glycogenin (*Gyg*), the core protein required for the initiation of glycogenesis, was induced 4.1-fold while losing its oscillating pattern. Additionally, 3.1-fold induction of UDP-glucose pyrophosphorylase 2 (*Ugp2*) was consistent with the 26.7-fold increase in hepatic levels of UDP-glucose, the activated monomer required for glycogen synthesis (Fig. 9). Continuous induction of *Gyg* and *Ugp2* may be an attempt to restore depleted glycogen levels. The inability to store glucose during the feeding phase would compromise energy availability during fasting and limit optimal nutrient utilization. This, combined with impaired gluconeogenesis, suggests TCDD disrupted circadian regulation of carbohydrate metabolism, consistent with reports of lower circulating glucose levels and altered glucose tolerance in TCDD-treated mice^31,65^.

### Heme biosynthesis

**Figure 10:**
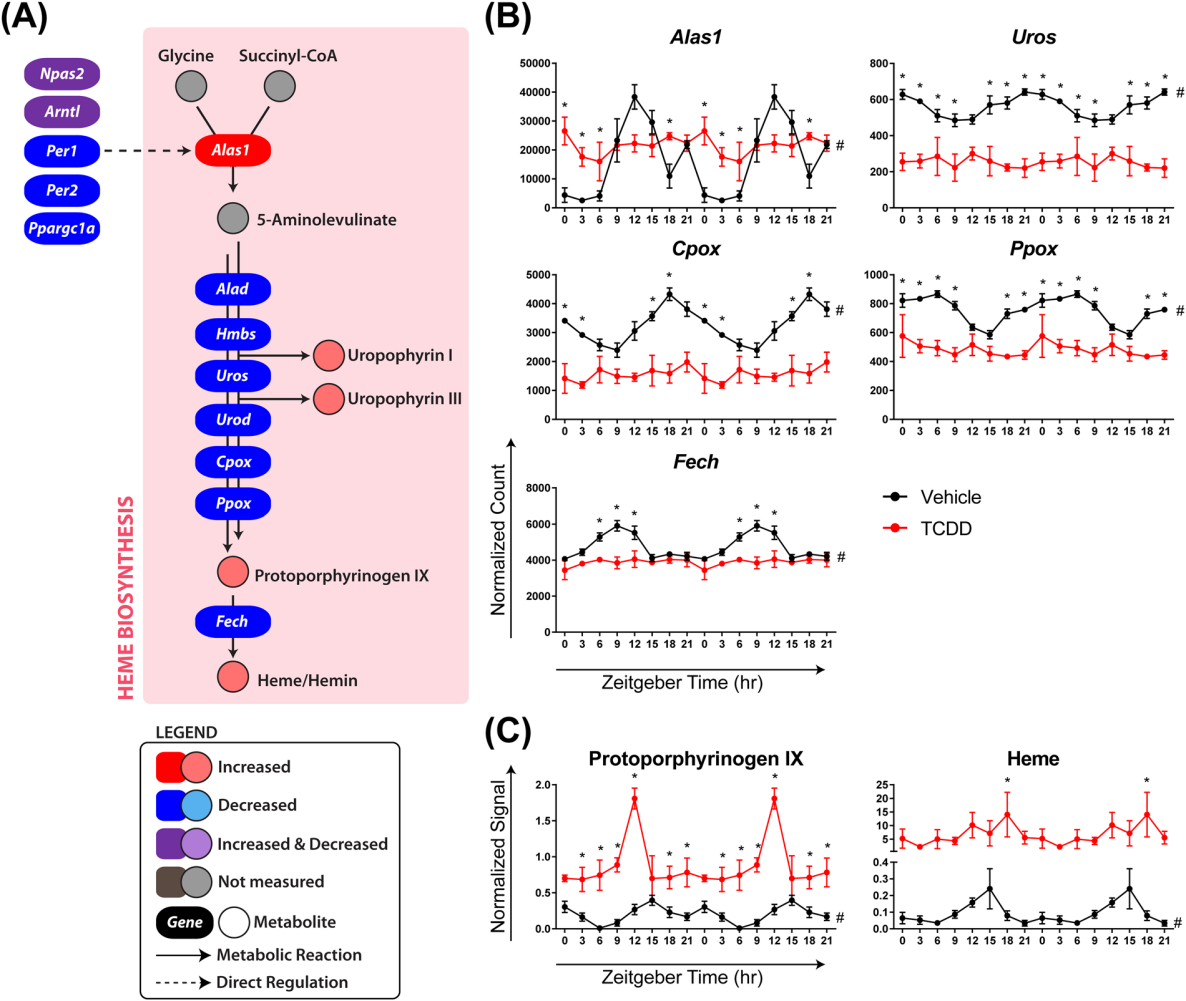
TCDD disrupts the circadian regulation of heme biosynthesis. Male C57BL/6 mice were orally gavaged with sesame oil vehicle or 30 µg/kg TCDD every 4 days for 28 days. (**A**) The effect of TCDD on hepatic heme biosynthesis. TCDD-elicited changes in hepatic (**B**) genes and (**C**) metabolites involved in heme biosynthesis. For genes, data points represent the average of 3 individual mice ± standard error of the mean (SEM), where posterior probabilities (*P1(t) ≥ 0.80) comparing vehicle and TCDD were determined using an empirical Bayes method. For metabolites, data points represent the average of 3–5 individual mice ± SEM, where statistical significance (*p ≤ 0.05) between vehicle and TCDD was determined using a 2-way ANOVA analysis followed by Sidak’s multiple comparison test. Diurnal rhythmicity was assessed using JTK_CYCLE (# BH q ≤ 0.1). Data are double-plotted along the x-axis for better visualization of rhythmicity.

Heme regulates circadian cycling by serving as a cofactor for REV-ERBα/β, NPAS2, CLOCK, and PER2^34–37^, and in turn, several key clock components regulate heme biosynthesis. Specifically, NPAS2, ARNTL, PER1, and PER2 regulate the rhythmic expression of aminolevulinic acid synthase 1 (*Alas1*), the rate-limiting step in hepatic heme biosynthesis^66^. In accordance with the diminished rhythmicity of *Npas2*, *Arntl*, *Per1*, and *Per2*, TCDD abolished the diurnal oscillation of *Alas1* and induced its expression 8.2-fold. As a result, hepatic protoporphyrinogen IX and heme levels were arrhythmic and increased 88.3- and 176.8-fold, respectively, following treatment (Fig. 10). *Alas1* is also controlled by PPAR coactivator 1α (PPARGC1A; aka PGC-1α), which is transcriptionally regulated by nutrient availability^67^. Consequently, heme serves as a signal of nutritional status, allowing the hepatic clock to respond to changes in nutrient availability. Interestingly, *Ppargc1a* was repressed 4.0-fold by TCDD, indicating the link between *Ppargc1a* and *Alas1* expression was disrupted. Therefore, heme levels no longer reflect nutrient availability, rendering the clock less responsive to nutritional status. Four of the seven genes downstream of *Alas1* in the heme biosynthesis pathway (*Uros*, *Cpox*, *Ppox*, and *Fech*) also lost their rhythmicity following TCDD treatment (Fig. 10). Overall, the effects of TCDD on heme biosynthesis would not only disrupt diurnal rhythmicity of the hepatic clock, but also impair entrainment with nutrient availability.

## Discussion

The current study investigated the effects of TCDD on the rhythmicity of the hepatic transcriptome and metabolome in mice. AhR activation by TCDD dampened the rhythmic expression of 15 hepatic core clock genes, involving either a decrease in amplitude or complete loss of oscillation. In turn, diurnal oscillations in core clock protein levels were abolished, while genomic binding of ARNTL in known target genes was reduced. This is consistent with previous studies demonstrating that AhR activation decreased the amplitude of core clock regulators (e.g. *Arntl*, *Per1*, and *Per2*) in the liver, ovaries, and bone marrow^28–30^, while AhR deficiency increased the amplitude of oscillation^19^. Impaired core clock cycling abolished the rhythmicity of virtually all circadian-controlled hepatic genes and polar metabolites according to JTK_CYCLE analysis. Moreover, disruption of the hepatic clock was also evident at the gross pathology level, where RLW was devoid of daily oscillations.

Hepatic ChIP-Seq analysis identified AhR enrichment 2 h after TCDD treatment in several core clock genes, suggesting AhR directly alters the clock’s transcriptional feedback loops^31^. Approximately half of these core clock genes exhibited AhR binding at sites lacking a pDRE, suggesting DRE-independent AhR signaling is involved in clock disruption^46^. βNF-activated AhR has been shown to interact with ARNTL in Hepa1c1c7 cells impairing ARNTL/CLOCK heterodimer formation at E-boxes within the *Per1* promoter^25^. Hypotheses that AhR heterodimerizes with ARNTL are not surprising given the sequence homology between ARNTL and AhR’s canonical partner ARNT^15,16^. In this study, targeted ChIP-PCR analysis confirmed co-binding of AhR, ARNTL, and CLOCK within *Per2* and *Nr1d1* loci at sites containing an E-box but no pDRE. However, AhR enrichment had no effect on ARNTL or CLOCK genomic binding, suggesting ARNTL/CLOCK heterodimerization was not impaired by TCDD at the regions examined. Alternatively, AhR binding near ARNTL and CLOCK may hinder co-activator recruitment, leading to repression of core clock genes, disruption of circadian regulation, and loss of rhythmicity across the transcriptome.

In addition to reduced transcriptional rhythmicity of the core clock regulators, TCDD altered cues that entrain the hepatic clock. The hepatic peripheral clock can be reset by specific dietary nutrients such as glucose and amino acids, allowing synchronization with feeding times^68–70^. Nutrient levels regulate *Ppargc1a* expression, which controls the transcription of several metabolic enzymes including *Alas1*, the rate-limiting step of heme biosynthesis^67^. Heme is a cofactor for REV-ERBα/β, NPAS2, CLOCK, and PER2, facilitating synchronization with nutrient availability^34–37^. TCDD abolished diurnal cycling of *Alas1* and downstream *de novo* heme biosynthesis gene expression, resulting in the accumulation and loss of rhythmicity in hepatic protoporphyrinogen IX and heme levels. Consequently, heme levels no longer reflected nutrient availability. In addition, previous studies report TCDD alters the segment-specific expression of intestinal nutrient transporters (e.g. fatty acid transporter *Cd36*, amino acid transporter *Slc36a1*, glucose transporter *Slc2a9*)^31,38^, which may interfere with rhythmic nutrient absorption. Intracellular redox homeostasis, including oxidative cycling of peroxiredoxins (PRDXs), thioredoxins (TXNs), nicotinamide adenine dinucleotide (NADH), and nicotinamide adenine dinucleotide phosphate (NADPH), also entrains the core clock. TCDD not only abolished *Prdx*, thioredoxin reductase (*Txnrd*), and NAD co-enzyme rhythmicity, but also disrupted circadian-regulated redox pathways including glutathione (GSH) biosynthesis and uric acid metabolism (Supplementary Information). This may alter the intracellular balance between pro- and anti-oxidants, further compromising hepatic clock entrainment.

AhR-mediated alterations in lipid, glucose, heme, bile acid, purine, and GSH metabolism contribute to TCDD-elicited NAFLD^31,32,46,65,71^. Here, we show TCDD disrupts the circadian regulation of these pathways, leading to the loss of diurnal rhythmicity in hepatic metabolite levels. As a result, synchronization between hepatic metabolism, energy homeostasis, and nutrient utilization may be compromised, despite no change in total daily food consumption. Previous studies also report TCDD has no effect on food consumption^54^, while others show decreased consumption after high dose administration does not account for weight loss and altered hepatic metabolism^72,73^. Taken together, this suggests TCDD-induced wasting and hepatotoxicity may involve inefficient nutrient metabolism and the inability to store energy. More specifically, the inhibition of glycogenesis and beta oxidation would limit energy availability during fasting. Meanwhile, central carbon intermediates are redirected to sustain heme and GSH biosynthesis, further compromising energy storage activities during nutrient-rich periods.

Although total food intake was unaffected by TCDD, feeding rhythmicity was not monitored in this study and therefore synchronization of circadian behaviors was not directly verified. If the global loss of rhythmicity was an artefact of asynchronization between TCDD-treated mice, RNA-Seq read count variation for circadian-regulated genes would be expected to be greater in TCDD-treated mice compared to vehicle controls^74^. Linear regression analysis of the COV for each circadian-regulated gene revealed that variation in gene expression was roughly equivalent between control and treated mice, suggesting the mice were not asynchronously rhythmic. Moreover, JTK_CYCLE analysis identified 23 genes which maintained rhythmicity following TCDD treatment including 11 core clock genes, providing further evidence that the TCDD-treated mice were synchronized. Based on these analyses, it was concluded that TCDD caused arrhythmicity in each mouse rather than asynchronous rhythmicity between mice.

Increased AhR binding within hepatic core clock genes, together with differential expression of core clock genes as early as 4 h after treatment, provides compelling evidence that TCDD directly disrupts the hepatic clock’s transcriptional feedback loops. Interestingly, TCDD also alters the rhythmicity of other peripheral clocks (ovaries, bone marrow)^29,30^, as well as the SCN master pacemaker itself ^26,27^, suggesting AhR-mediated clock disruption is not unique to the liver. Although the rhythmic expression of most hepatic genes is regulated by the local molecular clock, a subset are driven by systemic oscillating cues originating in the SCN^57,58^. TCDD-elicited alterations in SCN-derived systemic cues and circadian behaviors (i.e. sleep/wake and feeding/ fasting cycles) may therefore have contributed to the observed collapse of the hepatic clock. The loss of rhythmicity in system-driven genes identified by Kornmann *et al*. is consistent with disrupted SCN cycling, although direct AhR binding within these genes may also be a factor.

Overall, disruption of hepatic circadian rhythmicity by TCDD likely involves: (i) AhR-mediated repression of hepatic core clock regulators, (ii) direct AhR genomic binding within circadian-regulated target genes, and (iii) disruption of SCN rhythmicity and systemic oscillating cues (e.g. feeding/fasting cycles, body temperature fluctuations, diurnal hormones). Interactions between the local hepatic oscillator and rhythmic systemic cues further complicate studies aiming to elucidate entrainment mechanisms and identify the primary targets affected by TCDD. Future studies using restricted feeding protocols in combination with conditional tissue-specific genetic models may distinguish direct AhR effects from indirect changes driven by systemic cues.

Beyond effects on hepatic metabolism, disruptions in circadian rhythmicity have been associated with changes in the gut microbiome, increased intestinal permeability, NAFLD, obesity, cancer, and accelerated aging^4,5,75–78^. Therefore, AhR-mediated dysregulation of the circadian clock may be a unifying mechanism which contributes to the pleiotropic effects of TCDD including hepatotoxicity, wasting syndrome, hepatocellular carcinoma, and gut dysbiosis. Additional studies are needed to determine if these effects extend to dietary and microbial AhR ligands, as well as the relevance of AhR-mediated dysregulation of circadian rhythm in humans.

## Supplementary information

Supplementary Information
