# Supplementary material for: 2,3,7,8-Tetrachlorodibenzo-p-dioxin abolishes circadian regulation of hepatic metabolic activity in mice
Source: Sci Rep. 2019 Apr 24;9:6514. doi: 10.1038/s41598-019-42760-3 (PMC6478849; doi:10.1038/s41598-019-42760-3)
Supplement: Supplementary file 1 — Supplementary Information [file 41598_2019_42760_MOESM1_ESM.pdf]

SUPPLEMENTARY INFORMATION

2,3,7,8-Tetrachlorodibenzo-*p*-dioxin abolishes circadian regulation of hepatic metabolic activity in mice

Kelly A. Fader[1,2], Rance Nault[1,2], Claire M. Doskey[1,2], Russell R. Fling[2,3], and Timothy R. Zacharewski[1,2,*]

[1]Department of Biochemistry & Molecular Biology, Michigan State University, East Lansing, MI, 48824

[2]Institute for Integrative Toxicology, Michigan State University, East Lansing, MI, 48824

[3]Department of Microbiology and Molecular Genetics, Michigan State University, East Lansing, MI, 48824

[*]Corresponding author:

Timothy R. Zacharewski, Ph.D.

Michigan State University

603 Wilson Road, Room 309

East Lansing, MI, 48824-1319

Telephone: 517 – 355 – 1607

E-mail: tzachare@msu.edu

SUPPLEMENTARY RESULTS

Bile acid homeostasis

Several genes associated with bile acid biosynthesis and transport are circadian-regulated, allowing bile homeostasis to be synchronized with lipid consumption. For example, hepatic transcription of *Cyp7a1*, the rate-limiting step of primary bile acid biosynthesis, is regulated by REV-ERBα, DBP, and NFIL3 [1,2]. In turn, daily fluctuations in the concentration and composition of the hepatic and serum bile acid pools influence the rhythmicity of bile acid metabolizing genes through farnesoid X receptor (FXR) signaling [3]. In agreement with dampened *Nr1d1*, *Dbp*, and *Nfil3* oscillations, TCDD repressed hepatic *Cyp7a1* 38.8-fold and abolished its rhythmic expression. Several hepatic bile acid transporters also lost rhythmicity and were repressed including blood-to-liver importer *Slc10a1* and liver-to-bile canaliculus exporter *Abcb11* (31.3- and 7.0-fold, respectively), while the liver-to-blood exporter *Abcc4* was induced 134.9-fold (Supplementary Figure S7). These disruptions in enterohepatic circulation are consistent with the observed hepatic accumulation and loss of circadian oscillations in several bile acids including a primary taurine conjugate (3.8-fold), a secondary taurine conjugate (not-significant), and a cholic acid derivative (225.4-fold) (Supplementary Figure S7), similar to previous studies [4,5]. This loss of circadian regulation of bile acid homeostasis is further evidence of the decoupling between nutrient absorption, hepatic metabolism, and nuclear receptor signaling.

Redox homeostasis

The antioxidant proteins peroxiredoxin (PRDX) and thioredoxin (TXN), which exhibit daily oscillations in their oxidation states, are part of the common circadian ancestral system conserved across all domains of life [6,7]. Interestingly, abolishment of the PRDX clock system in *S. elongatus* and *A. thaliana* alters the amplitude or phase of core clock gene expression, demonstrating an intertwined yet poorly understood relationship between PRDX cycling and the clock's transcriptional feedback loops [6]. TCDD flattened and repressed hepatic expression of peroxiredoxin 4 (*Prdx4*; 2.9-fold) and 6 (*Prdx6*; 2.0-fold). Additionally, the predominant hepatic thioredoxin reductase *Txnrd1* was persistently induced 3.0-fold, while *Txnrd2* and *Txnrd3* were arrhythmic and repressed (4.0- and 5.3-fold respectively) following treatment (Supplementary Figure S8). In mammals, intracellular redox status also directly regulates the core clock feedback loops, where the reduced forms of nicotinamide adenine dinucleotide (NADH) and nicotinamide adenine dinucleotide phosphate (NADPH) enhance the DNA-binding activity of the ARNTL/CLOCK and ARNTL/NPAS2 heterodimers [8]. TCDD repressed (2.3-fold) and flattened

hepatic expression of nicotinamide phosphoribosyltransferase (*Nampt*), which catalyzes the rate-limiting conversion of nicotinamide (NAM) to nicotinamide mononucleotide (NMN). This led to loss of rhythmicity in hepatic NAM and NAD^+ levels. In addition, TCDD reduced hepatic NADPH levels 12.0-fold and abolished its oscillations (Supplementary Figure S8). Perturbations in NAD coenzyme rhythmicity may compromise the redox-regulated entrainment of the core clock machinery. However, further studies investigating TCDD-elicited changes in the cyclic ratios of the oxidized to reduced forms (i.e. NAD^+:NADH; $NADP^+$:NADPH) are required.

Given that redox status entrains circadian rhythmicity, it is not surprising that the circadian clock regulates reactive oxygen species (ROS) homeostasis and antioxidant responses such as glutathione (GSH) biosynthesis [7]. GSH is synthesized from cysteine, glutamate, and glycine in a two-step enzymatic process catalyzed by glutamate-cysteine ligase (*Gclc*) and glutathione synthetase (*Gss*), both of which are circadian regulated. TCDD persistently induced *Gclc* and *Gss* 4.8- and 3.8-fold, respectively, abolishing their rhythmicity. Glutathione reductase (*Gsr*), responsible for recycling oxidized glutathione disulfide (GSSG) back to GSH, also lost rhythmicity and was induced 6.2-fold (Supplementary Figure S9). The sustained induction of *Gclc* and *Gss* is in accordance with a 7.0-fold increase and loss of rhythmicity in the GSH precursor γ-glutamyl-cysteine (Supplementary Figure S9). Moreover, independent studies by our group detected a 1.3-fold increase in total hepatic GSH (GSH and GSSG) following TCDD treatment [9], as well as a 3.4-fold increase in hepatic glutamate (Fling *et al*., in preparation). Although increased antioxidant capacity is advantageous in response to TCDD-elicited oxidative stress, de-synchronization between the peripheral circadian clock and redox homeostasis may compromise cell signaling and overall liver function.

Beyond GSH, redox homeostasis is also affected by levels of uric acid, a breakdown product of purine nucleosides. In plasma, uric acid is a strong reducing agent that scavenges ROS, while intracellular uric acid promotes oxidative stress and stimulates pro-inflammatory pathways [10]. Several circadian-controlled genes involved in uric acid metabolism were affected by TCDD. Specifically, TCDD abolished the oscillation of ecto-5'-nucleotidase (*Nt5e*; induced 21.9-fold) and cytosolic II 5'-nucleotidase (*Nt5c2*; repressed 1.8-fold), which catalyze the hydrolysis of xanthosine monophosphate (XMP) to xanthosine. Purine-nucleoside phosphorylase (*Pnp*), which converts xanthosine to xanthine, also lost its rhythmicity. The oxidation of xanthine by xanthine dehydrogenase/xanthine oxidase (*Xdh/Xo*) yields uric acid, which is further oxidized to allantoin by urate oxidase (*Uox*). *Xdh/Xo* was induced 1.4-fold by TCDD, consistent with previous reports that *Xdh/Xo* induction contributes to AhR-mediated oxidative stress [9,11]. TCDD also flattened *Uox* rhythmic oscillation, with a 35.0-fold reduction in expression. Accordingly,

hepatic levels of uric acid and its upstream metabolites xanthosine and xanthine were reduced 45.9-, 157.6-, and 5.6-fold by TCDD, while concurrently losing their circadian oscillations (Supplementary Figure S10). Overall, uric acid metabolism was shut down by TCDD, suggesting purine nucleosides may be shunted toward alternative metabolic pathways.

SUPPLEMENTARY FIGURES

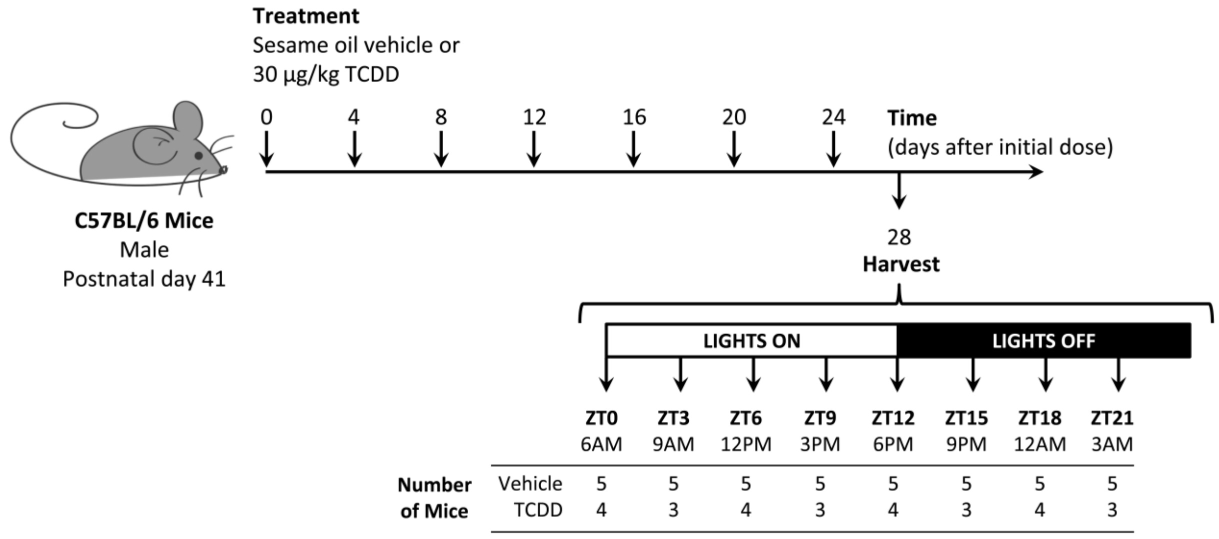

Supplementary Figure S1: Study design. Male C57BL/6 mice were orally gavaged with sesame oil vehicle or 30 µg/kg TCDD every 4 days for 28 days. Beginning at ZT0 on day 28, mice were euthanized every 3h for 24h (8 time points). The number of mice euthanized at each time point is shown.

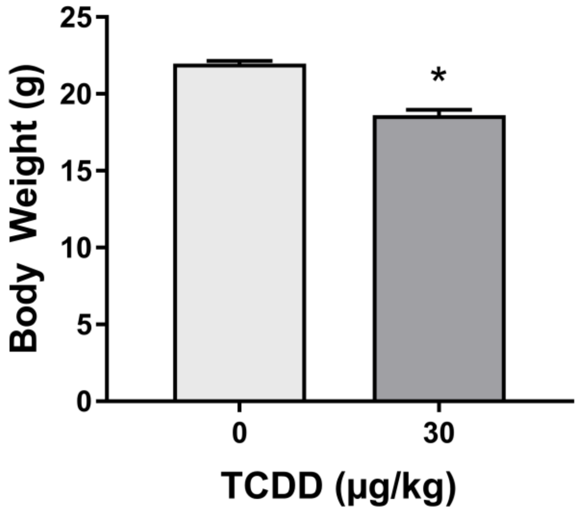

Supplementary Figure S2: The effect of TCDD on terminal body weight. Male C57BL/6 mice were orally gavaged with sesame oil vehicle or 30 µg/kg TCDD every 4 days for 28 days. Mice were weighed prior to euthanasia on day 28 of the study. Statistical significance (* p ≤ 0.05) was evaluated using a t-test.

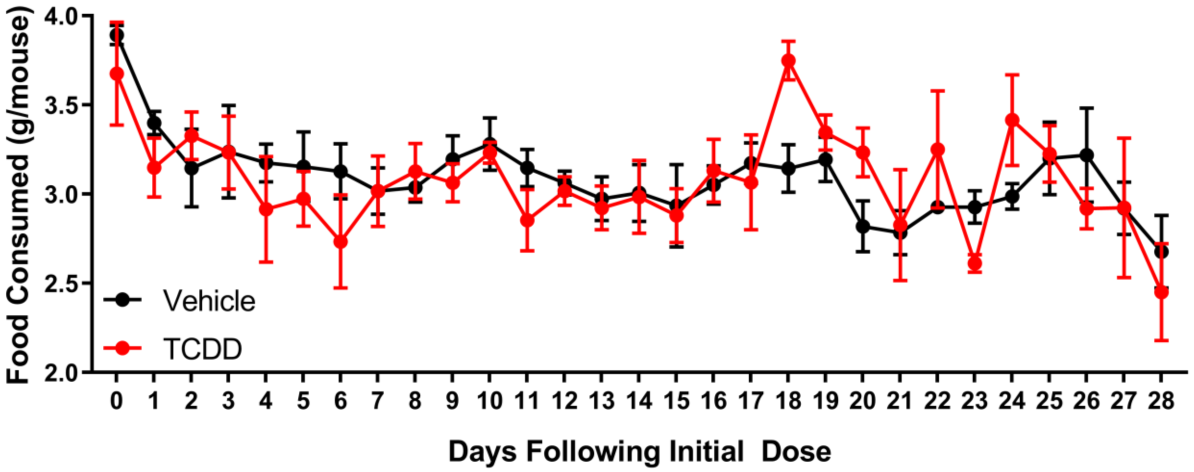

Supplementary Figure S3: TCDD had no effect on daily food consumption. Male C57BL/6 mice were orally gavaged with sesame oil vehicle or 30 µg/kg TCDD every 4 days for 28 days. The food trough for each cage of co-housed mice was weighed daily and food consumption was calculated based on the number of mice in the cage. Data points represent the average of 4 cages per treatment group ± standard error of the mean. Statistical significance ($p \leq 0.05$) was evaluated through a 2-way ANOVA analysis followed by Sidak's multiple comparison test.

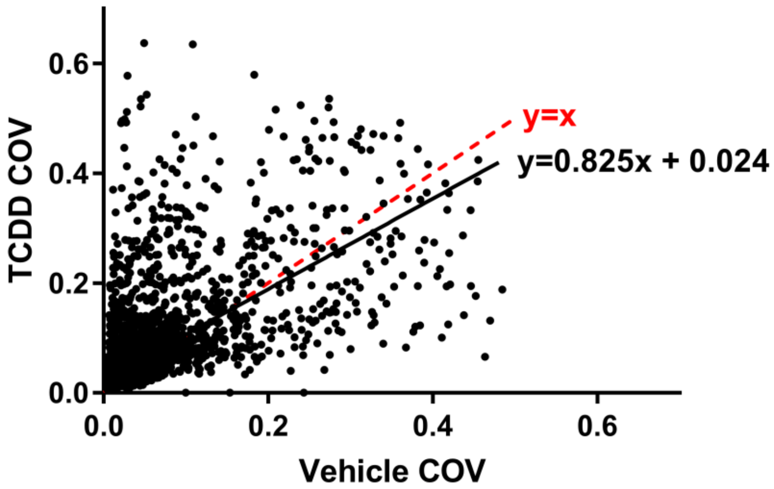

Supplementary Figure S4: Comparison of variation in circadian regulated gene-expression between vehicle and TCDD-treated mice. For each gene classified as rhythmic in controls, the coefficient of variation (COV) for the normalized $\log_2$-transformed read counts (n=3 mice) was determined at each timepoint. The average COV across the 8 timepoints was calculated for both controls and TCDD-treated mice. A total of 5636 genes are plotted, where each dot represents a single gene. The solid black line represents the results of the linear regression analysis, while the red dotted-line indicates the expected slope (equal to 1) if the variation between controls and TCDD-treated mice was equivalent.

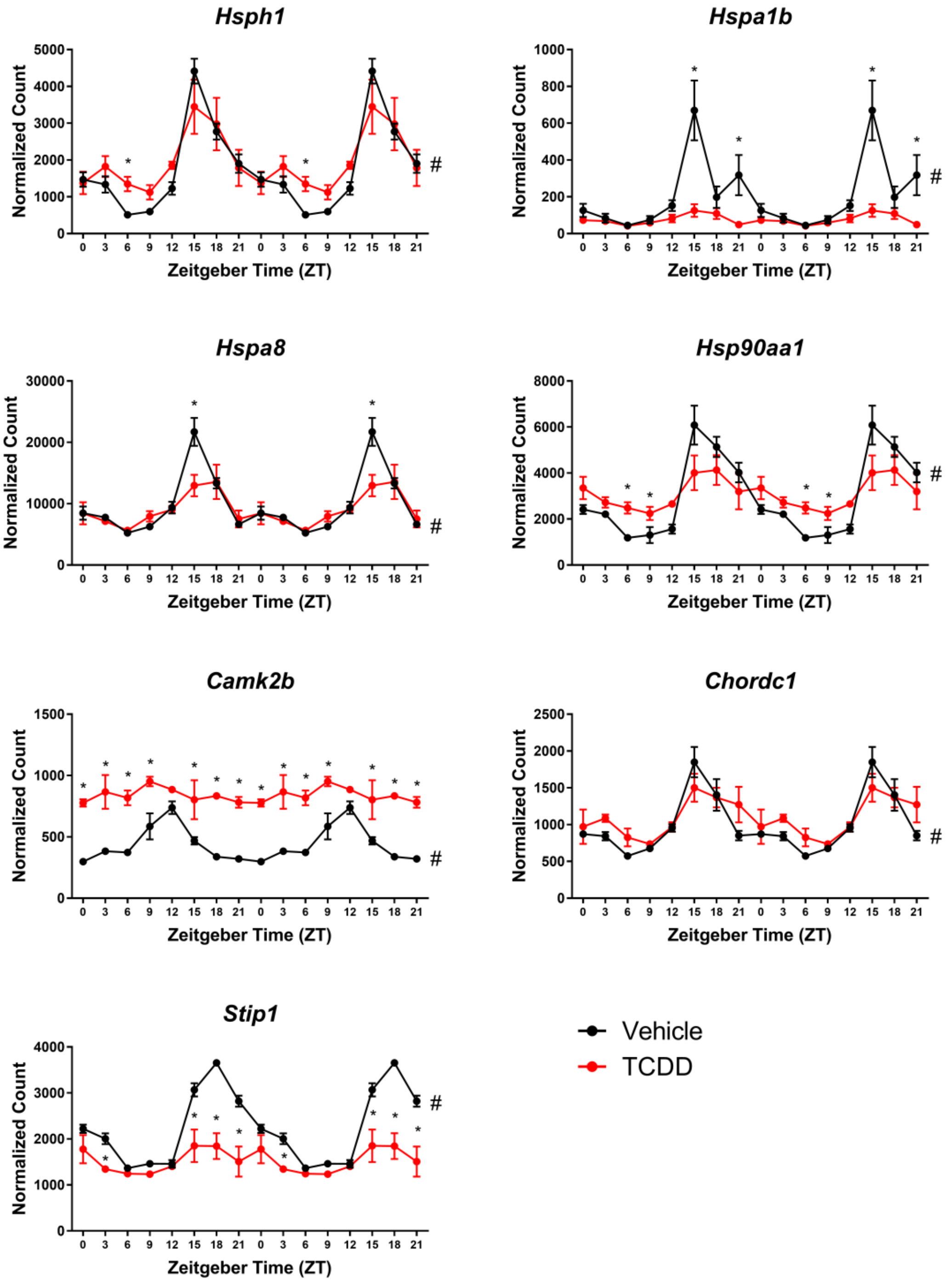

Supplementary Figure S5: System-driven genes which are regulated by heat shock transcription factor 1 (HSF1) and/or play a role in the heat shock response. Male C57BL/6 mice were orally gavaged with sesame oil vehicle or 30 µg/kg TCDD every 4 days for 28 days. Data points represent the average of 3 individual mice ± standard error of the mean (SEM), where posterior probabilities (* P1(t) ≥ 0.80) comparing vehicle and TCDD were determined using an empirical Bayes method. Diurnal rhythmicity was assessed using JTK_CYCLE (# BH q ≤ 0.1). Data are double-plotted along the x-axis for better visualization of rhythmicity.

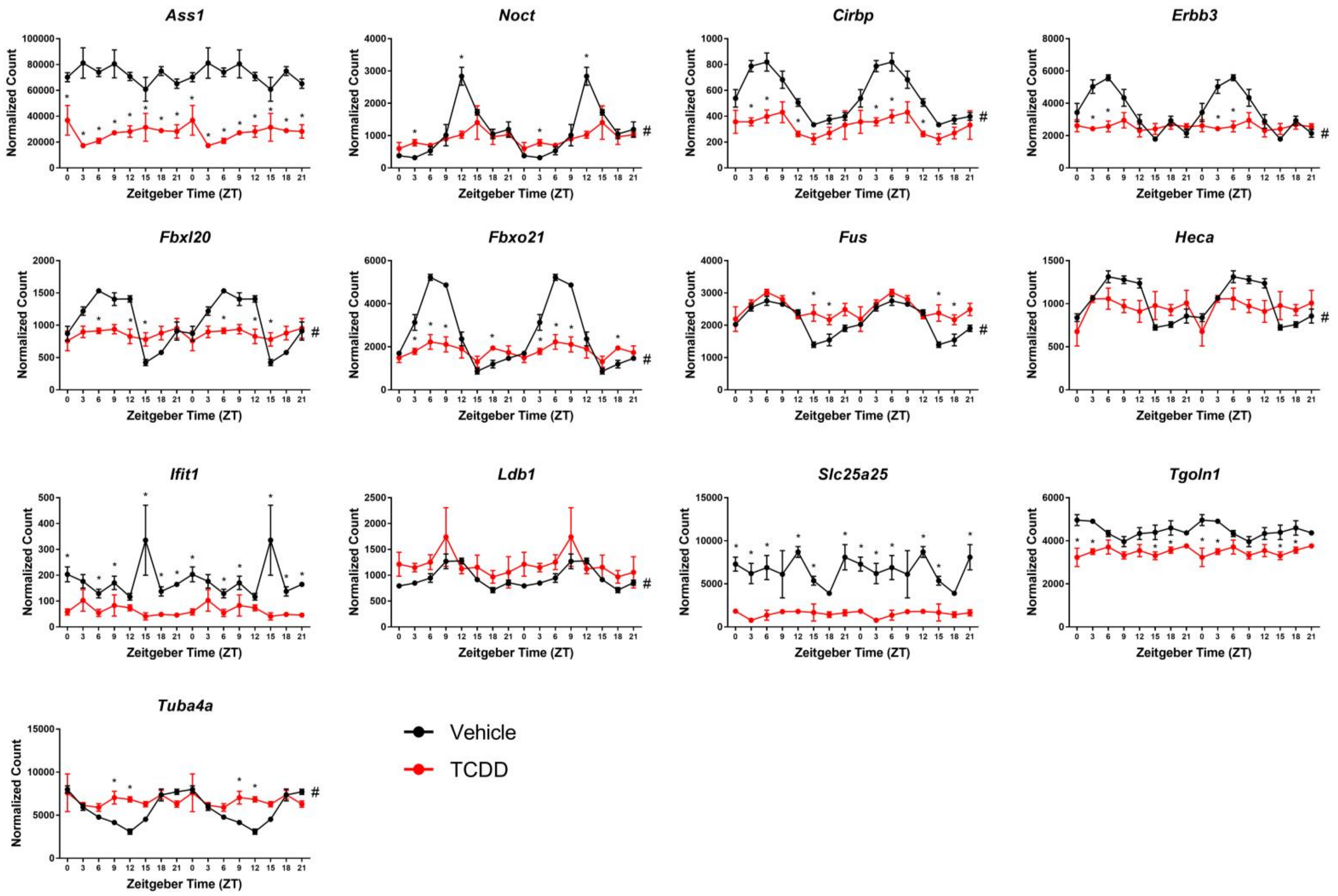

Supplementary Figure S6: System-driven genes which are not involved in the heat shock response. Male C57BL/6 mice were orally gavaged with sesame oil vehicle or 30 µg/kg TCDD every 4 days for 28 days. Data points represent the average of 3 individual mice ± standard error of the mean (SEM), where posterior probabilities (* P1(t) ≥ 0.80) comparing vehicle and TCDD were determined using an empirical Bayes method. Diurnal rhythmicity was assessed using JTK_CYCLE (# BH q ≤ 0.1). Data are double-plotted along the x-axis for better visualization of rhythmicity.

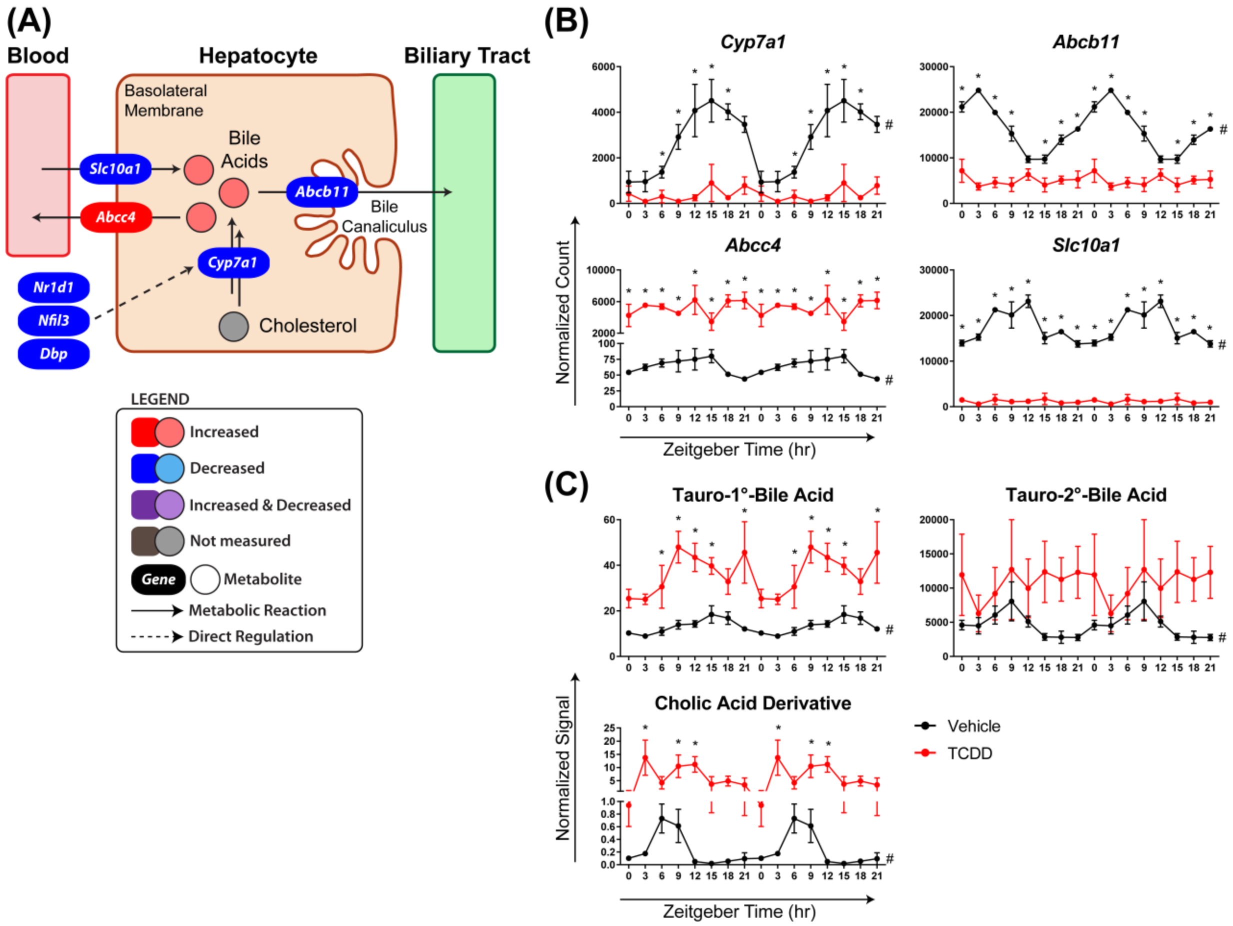

Supplementary Figure S7: TCDD disrupts circadian regulation of bile acid homeostasis. Male C57BL/6 mice were orally gavaged with sesame oil vehicle or 30 µg/kg TCDD every 4 days for 28 days. (A) The effect of TCDD on bile acid biosynthesis and hepatic transport. TCDD-elicited changes in hepatic (B) genes and (C) metabolites involved in bile acid homeostasis. For genes, data points represent the average of 3 individual mice ± standard error of the mean (SEM), where posterior probabilities (* $P1(t) \geq 0.80$) comparing vehicle and TCDD were determined using an empirical Bayes method. For metabolites, data points represent the average of 3-5 individual mice ± SEM, where statistical significance (* $p \leq 0.05$) between vehicle and TCDD was determined using a 2-way ANOVA analysis followed by Sidak's multiple comparison test. Circadian rhythmicity was assessed using JTK_CYCLE (# BH $q \leq 0.1$). Data are double-plotted along the x-axis for better visualization of rhythmicity.

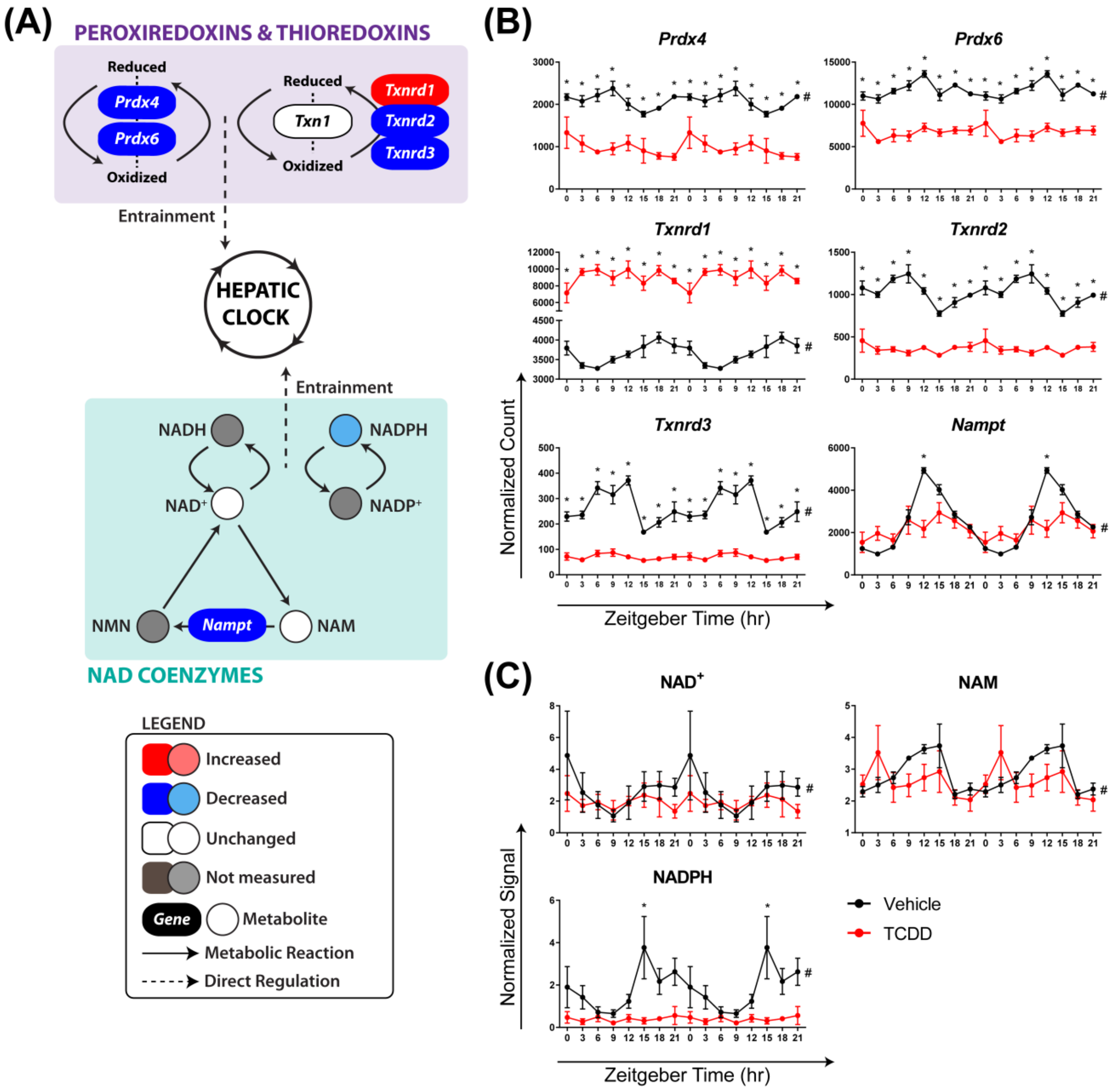

Supplementary Figure S8: TCDD disrupts redox-mediated entrainment of hepatic clock cycling. Male C57BL/6 mice were orally gavaged with sesame oil vehicle or 30 µg/kg TCDD every 4 days for 28 days. (A) The effect of TCDD on the peroxiredoxins (*Prdx*), thioredoxin reductases (*Txnrd*), and nicotinamide adenine dinucleotide (NAD) coenzymes. TCDD-elicited changes in hepatic (B) genes and (C) metabolites associated with the redox cycling of PRDX, TXN, and NAD coenzymes. For genes, data points represent the average of 3 individual mice ± standard error of the mean (SEM), where posterior probabilities (* P1(t) ≥ 0.80) comparing vehicle and TCDD were determined using an empirical Bayes method. For metabolites, data points represent the average of 3-5 individual mice ± SEM, where statistical significance (* p ≤ 0.05) between vehicle and TCDD was determined using a 2-way ANOVA analysis followed by Sidak's multiple comparison test. Circadian rhythmicity was assessed using JTK_CYCLE (# BH q ≤ 0.1). Data are double-plotted along the x-axis for better visualization of rhythmicity.

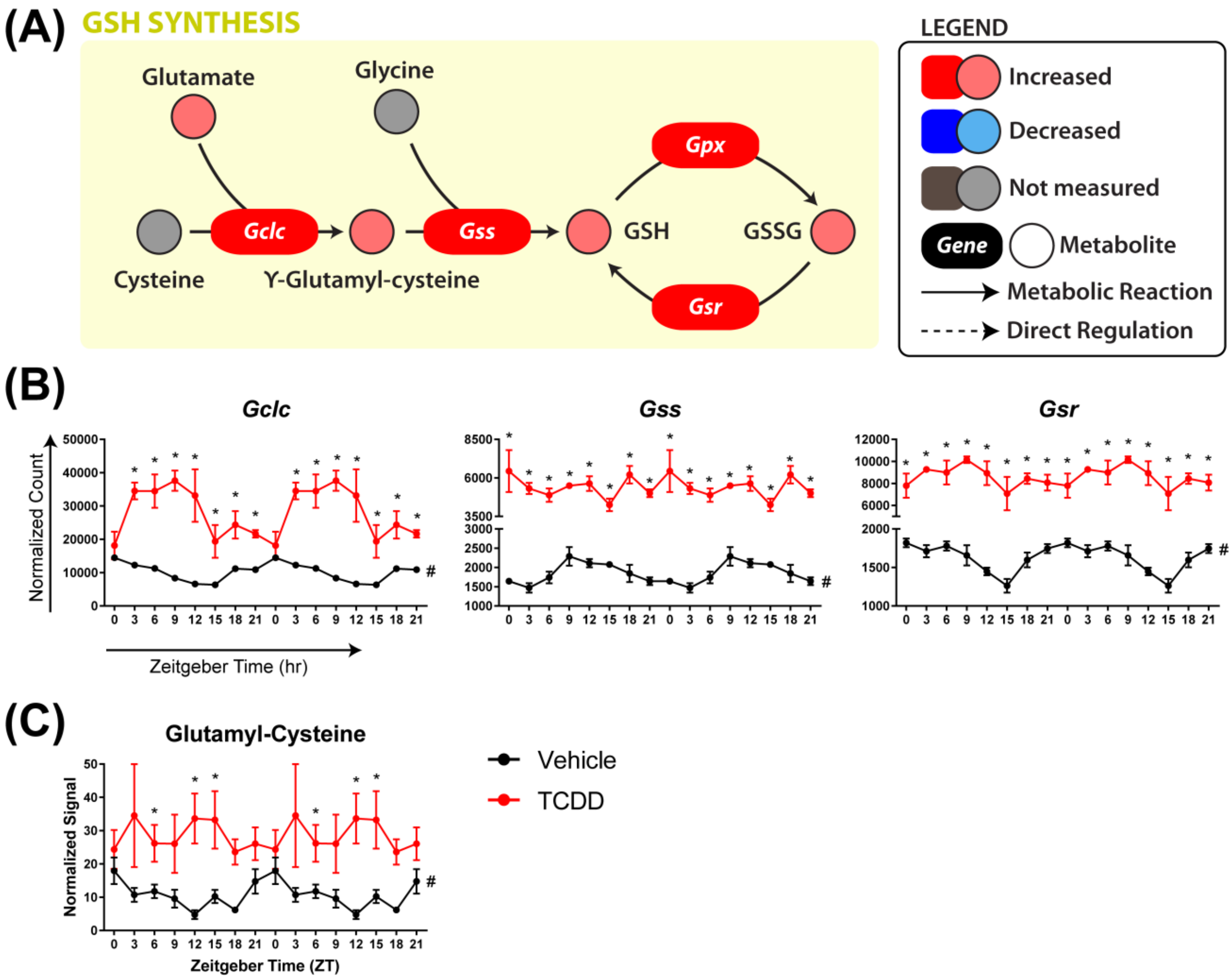

Supplementary Figure S9: TCDD disrupts circadian regulation of glutathione (GSH) homeostasis. Male C57BL/6 mice were orally gavaged with sesame oil vehicle or 30 µg/kg TCDD every 4 days for 28 days. (A) The effect of TCDD on GSH biosynthesis and recycling. TCDD-elicited changes in hepatic (B) genes and (C) metabolites involved in GSH biosynthesis. For genes, data points represent the average of 3 individual mice ± standard error of the mean (SEM), where posterior probabilities (* P1(t) ≥ 0.80) comparing vehicle and TCDD were determined using an empirical Bayes method. For metabolites, data points represent the average of 3-5 individual mice ± SEM, where statistical significance (* $p \leq 0.05$) between vehicle and TCDD was determined using a 2-way ANOVA analysis followed by Sidak's multiple comparison test. Circadian rhythmicity was assessed using JTK_CYCLE (# BH $q \leq 0.1$). Data are double-plotted along the x-axis for better visualization of rhythmicity.

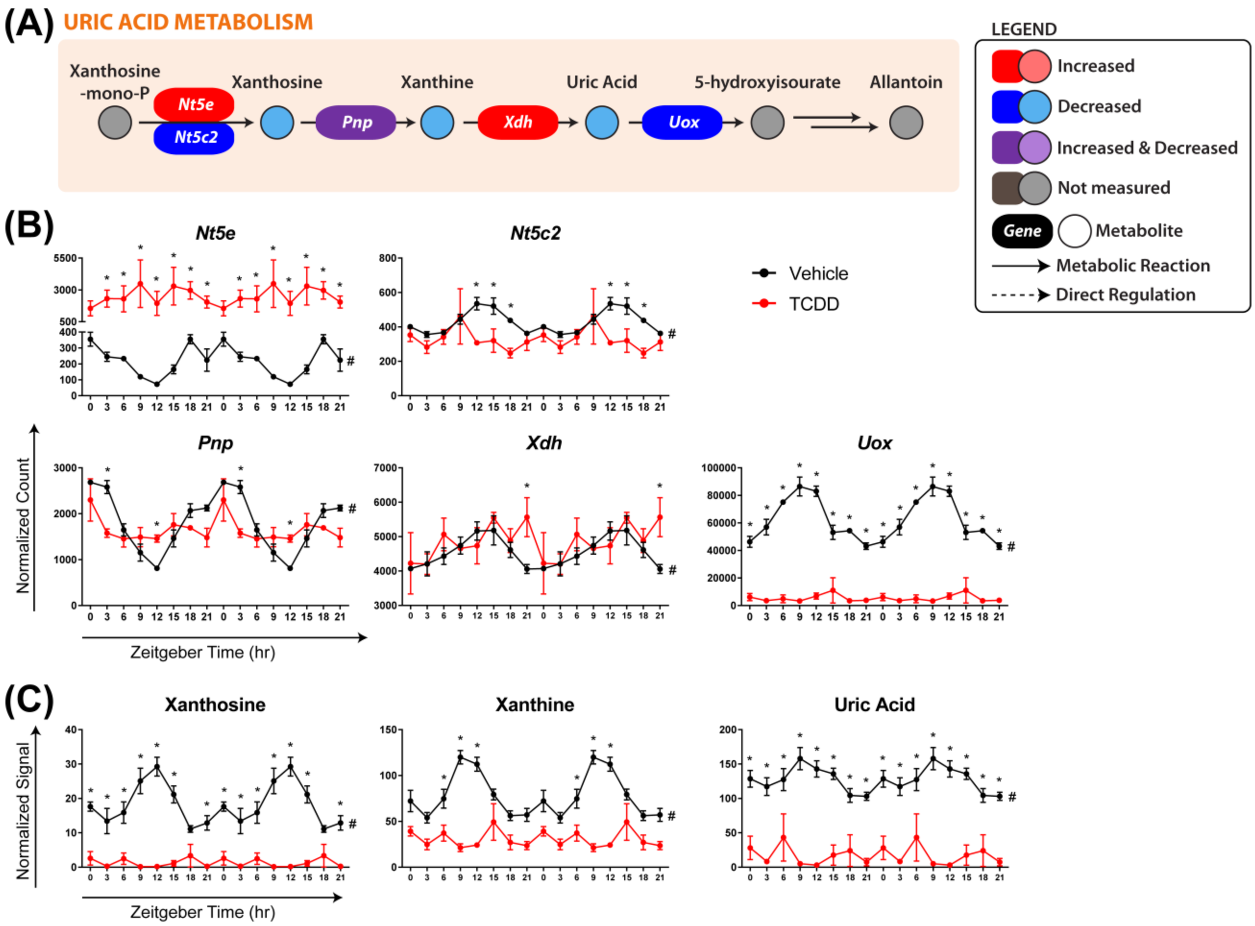

Supplementary Figure S10: TCDD disrupts circadian regulation of uric acid metabolism. Male C57BL/6 mice were orally gavaged with sesame oil vehicle or 30 µg/kg TCDD every 4 days for 28 days. (A) The effect of TCDD on purine metabolism and uric acid biosynthesis. TCDD-elicited changes in hepatic (B) genes and (C) metabolites involved in uric acid metabolism. For genes, data points represent the average of 3 individual mice ± standard error of the mean (SEM), where posterior probabilities (* P1(t) ≥ 0.80) comparing vehicle and TCDD were determined using an empirical Bayes method. For metabolites, data points represent the average of 3-5 individual mice ± SEM, where statistical significance (* p ≤ 0.05) between vehicle and TCDD was determined using a 2-way ANOVA analysis followed by Sidak's multiple comparison test. Circadian rhythmicity was assessed using JTK_CYCLE (# BH q ≤ 0.1). Data are double-plotted along the x-axis for better visualization of rhythmicity.

SUPPLEMENTARY TABLES

Supplementary Table S1: Primer sequences (5' -3') and product sizes for genes analyzed by qRT-PCR.

Gene ID	Symbol	Ref Seq	Forward Primer	Reverse Primer	Product Size
11461	*Actb*	NM_007393	GCTACAGCTTCACCACCACA	TCTCCAGGGAGGAAGAGGAT	123
14433	*Gapdh*	NM_008084	GTGGACCTCATGGCCTACAT	TGTGAGGGAGATGCTCAGTG	125
13076	*Cyp1a1*	NM_009992	AAGTGCAGATGCGGTCTTCT	AAAGTAGGAGGCAGGCACAA	140
11865	*Arntl*	NM_007489	GATGACCCTCATGGAAGGTTAGA	ATTTTGTCCCGACGCCTCTT	101
18143	*Npas2*	NM_008719	AGAGGCAGCTTGAACCCAAA	GAGGGGCTAGGCACATTGTT	92
18030	*Nfil3*	NM_017373	CAGGGAGCAGAACCACGATA	CCTACAGACCGGATGGAGGA	154
217166	*Nr1d1*	NM_145434	CTCGTCTCCCTCAGCCATTG	ACACCACCTGTGTTGTTATTGG	139
13170	*Dbp*	NM_016974	GCTGTCTCCTGAAGGAAAAGGAG	GGGACCCACCGCCACTAAC	123
21685	*Tef*	NM_017376	TGTCCAGCACAGAATCGTCC	GCAGGGTCAGGGTTGAAGTT	100

Supplementary Table S2: Primer sequences (5' -3') for genomic regions analyzed by ChIP-PCR.

Gene	Amplicon Coordinates	Amplicon Location	Forward Primer	Reverse Primer	Amplicon Size (bp)	pDRE?	E-box?	Reference
Per1	chr11:69,098,654-69,098,858	Promoter (Site 1)	ATCCTCCCTGAAAAGGGGTA	GGATCTCTTCCTGGCATCTG	205	No	Yes, within amplicon	Xu et al., 2010[12]
Per1	chr11:69,094,904-69,094,974	Promoter (Site 2)	AGCCCTCTCAGCCTATGAGAAAGT	CCCGCCCTGCCTAAATCA	71	Yes, near amplicon	Yes, near amplicon	Rey et al., 2011[13]
Per2	chr1:91,459,371-91,459,448	Promoter	TCATTTGCATACTGGCGGGG	TATGTAAAGAGAGCGACGGGC	78	No	Yes, near amplicon	Designed based on unpublished ARNTL ChIP-Seq
Per2	chr1:91,446,265-91,446,397	Within intron 4	TCCGCCTTCACAGACTGGTA	ACCCTCCAGGGACTATTGGT	133	Yes, within amplicon	No	Designed based on AhR ChIP-Seq
Dbp	chr7:45,707,643-45,707,707	Within intron 2	TGGGACGCCTGGGTACAC	GGGAATGTGCAGCACTGGTT	65	Yes, near amplicon	Yes, near amplicon	Rey et al., 2011[13]
Nr1d1	chr11:98,783,446-98,783,535	Promoter	TGCAGCCTGCTCCATTTCTA	CCCCTCACTTGCACATGTCT	90	No	Yes, near amplicon	Designed based on unpublished ARNTL ChIP-Seq
Cyp1a1	chr9:57,696,696-57,696,767	Promoter	GAGGATGGAGCAGGCTTACG	GGGCTACAAAGGGTGATGCTT	72	Yes, near amplicon	No	Designed based on AhR ChIP-Seq
Negative Control	chr6:120,257,680-120,257,772	N/A	CTGGGGTCACCTACATCAGC	GAGCGAGATGATTCCTGCCA	93	No	No	Designed based on AhR and ARNTL ChIP-Seq

Supplementary Table S3: The effect of TCDD on the acrophase of rhythmic genes.

Gene Symbol	Vehicle Phase (h)	TCDD Phase (h)	Phase Shift (h; TCDD-VEH)
Rorc	18	24	6
Tef	10.5	15	4.5
Leprotl1	0	3	3
Rtel1	6	9	3
Ddo	16.5	19.5	3
Cry1	21	22.5	1.5
Nr1d1	7.5	9	1.5
Nr1d2	10.5	12	1.5
Slc39a10	18	19.5	1.5
Per3	12	13.5	1.5
Mios	7.5	9	1.5
Arntl	0	1.5	1.5
Npas2	0	0	0
Bclaf1	7.5	7.5	0
Nfil3	0	0	0
Dbp	10.5	10.5	0
Ddx46	10.5	9	-1.5
Hnrnpdl	9	7.5	-1.5
St5	1.5	0	-1.5
Per2	16.5	15	-1.5
Kpna2	15	10.5	-4.5
Polr1b	16.5	10.5	-6
Max	15	9	-6

REFERENCES

1 Lavery, D. J. & Schibler, U. Circadian transcription of the cholesterol 7 alpha hydroxylase gene may involve the liver-enriched bZIP protein DBP. *Genes Dev* **7**, 1871-1884 (1993).

2 Duez, H. *et al.* Regulation of bile acid synthesis by the nuclear receptor Rev-erbalpha. *Gastroenterology* **135**, 689-698, doi:10.1053/j.gastro.2008.05.035 (2008).

3 Zhang, Y. K., Guo, G. L. & Klaassen, C. D. Diurnal variations of mouse plasma and hepatic bile acid concentrations as well as expression of biosynthetic enzymes and transporters. *PLoS One* **6**, e16683, doi:10.1371/journal.pone.0016683 (2011).

4 Fader, K. A. *et al.* 2,3,7,8-Tetrachlorodibenzo-p-dioxin (TCDD)-elicited effects on bile acid homeostasis: Alterations in biosynthesis, enterohepatic circulation, and microbial metabolism. *Sci Rep* **7**, 5921, doi:10.1038/s41598-017-05656-8 (2017).

5 Ma, K. *et al.* Circadian dysregulation disrupts bile acid homeostasis. *PLoS One* **4**, e6843, doi:10.1371/journal.pone.0006843 (2009).

6 Edgar, R. S. *et al.* Peroxiredoxins are conserved markers of circadian rhythms. *Nature* **485**, 459-464, doi:10.1038/nature11088 (2012).

7 Stangherlin, A. & Reddy, A. B. Regulation of circadian clocks by redox homeostasis. *J Biol Chem* **288**, 26505-26511, doi:10.1074/jbc.R113.457564 (2013).

8 Rutter, J., Reick, M., Wu, L. C. & McKnight, S. L. Regulation of clock and NPAS2 DNA binding by the redox state of NAD cofactors. *Science* **293**, 510-514, doi:10.1126/science.1060698 (2001).

9 Nault, R. *et al.* Pyruvate Kinase Isoform Switching and Hepatic Metabolic Reprogramming by the Environmental Contaminant 2,3,7,8-Tetrachlorodibenzo-p-Dioxin. *Toxicol Sci* **149**, 358-371, doi:10.1093/toxsci/kfv245 (2016).

10 Sautin, Y. Y. & Johnson, R. J. Uric acid: the oxidant-antioxidant paradox. *Nucleosides Nucleotides Nucleic Acids* **27**, 608-619, doi:10.1080/15257770802138558 (2008).

11 Sugihara, K. *et al.* Aryl hydrocarbon receptor (AhR)-mediated induction of xanthine oxidase/xanthine dehydrogenase activity by 2,3,7,8-tetrachlorodibenzo-p-dioxin. *Biochem Biophys Res Commun* **281**, 1093-1099, doi:10.1006/bbrc.2001.4464 (2001).

12 Xu, C. X., Krager, S. L., Liao, D. F. & Tischkau, S. A. Disruption of CLOCK-BMAL1 transcriptional activity is responsible for aryl hydrocarbon receptor-mediated regulation of Period1 gene. *Toxicol Sci* **115**, 98-108, doi:10.1093/toxsci/kfq022 (2010).

13 Rey, G. *et al.* Genome-wide and phase-specific DNA-binding rhythms of BMAL1 control circadian output functions in mouse liver. *PLoS Biol* **9**, e1000595, doi:10.1371/journal.pbio.1000595 (2011).
